# The evolving story of AtzT, a periplasmic binding protein

**DOI:** 10.1107/S2059798319013883

**Published:** 2019-10-31

**Authors:** Matthew L. Dennis, Lygie Esquirol, Tom Nebl, Janet Newman, Colin Scott, Thomas S. Peat

**Affiliations:** a Biomedical Manufacturing Program, CSIRO, 343 Royal Parade, Parkville, VIC 3052, Australia; b CSIRO Synthetic Biology Future Science Platform, GPO Box 1700, Acton, Canberra, ACT 2601, Australia

**Keywords:** periplasmic binding proteins, SAD phasing, atrazine, AtzT

## Abstract

Atrazine is an *s*-triazine-based herbicide that is used in many countries around the world in many millions of tons per year. A small number of organisms, such as *Pseudomonas* sp. strain ADP, have evolved to use this modified *s*-triazine as a food source, and the various genes required to metabolize atrazine can be found on a single plasmid. The atomic structures of seven of the eight proteins involved in the breakdown of atrazine by *Pseudomonas* sp. strain ADP have been determined by X-ray crystallography, but the structures of the proteins required by the cell to import atrazine for use as an energy source are still lacking. The structure of AtzT, a periplasmic binding protein that may be involved in the transport of a derivative of atrazine, 2-hydroxyatrazine, into the cell for mineralization, has now been determined. The structure was determined by SAD phasing using an ethylmercury phosphate derivative that diffracted X-rays to beyond 1.9 Å resolution. ‘Native’ (guanine-bound) and 2-hydroxyatrazine-bound structures were also determined to high resolution (1.67 and 1.65 Å, respectively), showing that 2-hydroxyatrazine binds in a similar way to the purportedly native ligand. Structural similarities led to the belief that it may be possible to evolve AtzT from a purine-binding protein to a protein that can bind and detect atrazine in the environment.

## Abstract 2

The structure of AtzT, which is thought to be involved in atrazine uptake in bacteria, was solved by SAD phasing using an ethylmercury phosphate derivative. Density in the binding site was subsequently found to be guanine that was retained by the protein throughout the purification process. Replacing the guanine with 2-hydroxyatrazine allowed the manner of binding of this substrate to be determined and its comparison with what appears to be the original ligand for this protein.

## 1. Introduction   

Atrazine is one of the most commonly used herbicides on the planet, with more than 70 million pounds used in the US alone every year (United States Environmental Protection Agency, 2013 ▸). While the use of atrazine has been reported to enable benefits to crop yields, there are significant concerns about its impact on the environment and human health (Nicolopoulou-Stamati *et al.*, 2016 ▸; Rickard *et al.*, 2018 ▸). Bacteria have evolved several pathways for the catabolism of atrazine and related synthetic compounds (*i.e.* other *s*-triazines), allowing them to convert these nitrogen-rich compounds to ammonia to support growth.

The best understood of the atrazine catabolism pathways is that from *Pseuodomonas* sp. strain ADP, in which a series of hydrolases (AtzA, AtzB, AtzC, AtzD, AtzEG, AtzH and AtzF) convert the heterocycle to ammonia and carbon dioxide *via* the metabolic intermediate cyanuric acid. The genes encoding AtzD, AtzEG, AtzH and AtzF, which are responsible for degrading cyanuric acid, are co-located in an operon on a self-transmissible plasmid (Udiković-Kolić *et al.*, 2012 ▸). A second operon, transcribed divergently from the *atzDGEHF* operon, contains genes that encode an ABC transporter (AtzS, AtzT, AtzU, AtzV and AtzW) and a cyanuric acid-responsive transcriptional regulator (AtzR) that regulates the transcription of both operons. Both operons are transcribed from σ^54^-dependent promoters and are induced under nitrogen starvation (Platero *et al.*, 2012 ▸). The *atzDGEHF* operon is further up-regulated by AtzR in the presence of cyanuric acid, while the *atzRSTUVW* operon is repressed by AtzR when cyanuric acid is present (Platero *et al.*, 2012 ▸).

The enzymes of the atrazine catabolic pathway are now biochemically and structurally well characterized (Peat *et al.*, 2013 ▸, 2015 ▸; Balotra, Warden *et al.*, 2015 ▸; Esquirol, Peat, Wilding, Liu *et al.*, 2018 ▸; Balotra, Newman *et al.*, 2015 ▸; Esquirol, Peat, Wilding, Hartley *et al.*, 2018 ▸), while the transport proteins encoded by the *atzRSTUVW* operon are not; however, there is some genetic evidence that these gene products are involved in the facilitated uptake of atrazine (Liu & Parales, 2009 ▸). For some bacteria, such as *Arthrobacter aurescens* TC1, mass transfer has been demonstrated to be the rate-limiting step in atrazine catabolism (Kundu *et al.*, 2019 ▸). Gaining an understanding of the mechanisms by which atrazine may be actively imported is therefore of high importance in understanding the dynamics of the process.

Periplasmic binding proteins (a subclass of solute-binding proteins, SBPs; Scheepers *et al.*, 2016 ▸) bind to a wide variety of small molecules and metals, and serve as a mechanism for sequestering these ligands for use by the organism. These proteins are part of a family of receptors that serve a variety of functions, for example transport, quorum sensing and chemotaxis. Additionally, some of these proteins have been engineered to be selective sensors for the identification of specific compounds in complex mixtures (Dwyer & Hellinga, 2004 ▸; Donaldson *et al.*, 2017 ▸; Newman *et al.*, 2019 ▸). The protein structures are generally conserved and have two α/β domains that bind the ligand of interest between the domains (often referred to as a ‘Venus fly-trap’ mechanism). These proteins can be found in both open and closed forms, where ligand binding shifts the equilibrium to the closed form, *i.e.* closing the fly trap (Felder *et al.*, 1999 ▸).

Here, we describe the characterization of the periplasmic binding protein AtzT, including high-resolution ‘closed’ structures with bound ligands. We also report the ligand preference of AtzT as determined by surface plasmon reson­ance (SPR).

## 2. Materials and methods   

### 2.1. Cloning, expression and purification   

The *atzT* gene, assigned as orf97 (NC_004956.1, 1440631) encoding protein Q936X6 in UniProt, was amplified from *Pseudomonas* sp. strain ADP genomic DNA. The restriction sites SacI and NdeI were inserted in the 5′ primer (5′→3′) catcatGAGCTCCATATGcaggaaccgctgaaagtcgcc and the restriction sites AvrII and BamHI were inserted in the 3′ region (5′→3′) catcatGGATCCTAGGtcacttcgggatcgtgccttcgac. Following amplification, the amplified gene portion was sub­cloned into pETcc2 vector using NdeI and BamHI (Supplementary Fig. S1).

The expression vector pETcc2-*atzT* was used to transform *Escherichia coli* BL21 (λDE3) cells (Invitrogen). The bacteria were grown on Luria–Bertani medium containing ampicillin (100 µg ml^−1^) for the pETcc2 constructs. The cells were grown with shaking at 200 rev min^−1^ at 301 K. Protein expression was induced at an OD_600_ of 0.8 by the addition of isopropyl β-d-1-thiogalactopyranoside (1 m*M* final concentration). A 1 l culture produced around 100 mg of AtzT.

The cells were harvested after 24 h of expression by centrifugation at 5000*g* for 15 min with an Avanti J-E centrifuge (Beckman Coulter, Indianapolis, USA), resuspended in lysis buffer (25 m*M* potassium phosphate, 5 m*M* imidazole pH 7.5) and lysed by passage through a Microfluidics M-110P homogenizer (Massachusetts, USA) five times at 103 MPa. The lysis was followed by centrifugation at 18 000*g* for 45 min to pellet the cellular debris, and the soluble fraction was used for further purification.

The soluble fraction was syringe-filtered through a 0.22 µm filter. The filtrate was purified using a 5 ml Ni–NTA Superflow cartridge (Qiagen, Maryland, USA) with a gradient from 5 to 500 m*M* imidazole over 20 column volumes. SDS–PAGE gel analysis was performed to assess the purity of the fractions. AtzT was found to elute at an imidazole concentration of between 130 and 300 m*M* (Supplementary Fig. S2). The fractions containing AtzT were pooled and concentrated to 12 ml using an Amicon Ultra-15 centrifugal filter unit. Finally, size-exclusion chromatography was performed using a 130 ml column packed with Superdex 75 preparation-grade resin (GE Healthcare Life Sciences) equilibrated with 50 m*M* HEPES pH 7.5, 200 m*M* NaCl over 1.5 column volumes (Supplementary Fig. S1). All chromatographic steps were performed using an ÄKTApurifier UPC 10 (GE Healthcare Life Sciences).

### 2.2. Crystallography and structure determination   

Crystallization was performed with the protein sample at 20 mg ml^−1^ (in 50 m*M* HEPES pH 7.5, 200 m*M* NaCl) in SD2 sitting-drop plates (Molecular Dimensions, UK) incubated at 293 K. The experimental droplets consisted of 150 nl protein sample plus 150 nl crystallization condition and were equilibrated against 50 µl crystallization condition. The crystals grew in conditions that consisted of 6–8%(*v*/*v*) Jeffamine M600 with 2–7%(*v*/*v*) MPD and trisodium citrate at 1.1–1.2 *M*. The crystals grew as flat plates with varying degrees of thickness, and would appear occasionally after 5–7 days without microseeding or reliably overnight with microseeding (see Supplementary Fig. S3). The crystals were harvested into MiTeGen Mylar loops and cryoprotected by the addition of MPD to give a 20%(*v*/*v*) final concentration. Diffraction data were collected at 100 K on the MX2 microfocus beamline at the Australian Synchrotron using a Dectris EIGER 16M detector (Aragão *et al.*, 2018 ▸). 360° of data were taken in 36 s to obtain full data sets. For the ethylmercury phosphate derivative, the wavelength was moved to 12 300 eV (1.0082 Å) and four full data sets were obtained from a single crystal by moving down the length of the crystal. Three of these data sets were shown to be of good quality and high resolution and were merged to obtain a highly redundant data set for SAD phasing (see Table 1 ▸ for X-ray data-set statistics). Data were indexed using *XDS* (Kabsch, 2010 ▸) and scaled using *AIMLESS* (Evans & Murshudov, 2013 ▸), and the structure was phased using *CRANK*2 (Skubák & Pannu, 2013 ▸), which gave an initial model with four protomers in the asymmetric unit and four Hg atoms. Owing to a mix-up in the clones (two different proteins of approximately equal size were being investigated at the same time), an incorrect sequence was originally input into *CRANK*2. A sequence of eight amino acids in clear electron density was used to search for the correct sequence in UniProt. The most likely sequence containing these eight residues was found to be UniProt ID Q936X6. This sequence was defined as being from *Pseudomonas* sp. strain ADP and was identified as AtzT, which was one of the two proteins under investigation. A model with the correct sequence was built manually using *Coot* (Emsley *et al.*, 2010 ▸) and refined using *REFMAC* (Murshudov *et al.*, 2011 ▸). Standard (default) *REFMAC* parameters were used for refinement with jelly-body refinement, isotropic *B* factors and automatic NCS restraints applied. Subsequent structures were phased using *Phaser *(McCoy *et al.*, 2007 ▸) and the initial SAD-based model, manually rebuilt using *Coot* and refined using *REFMAC*. *phenix.elbow* (Moriarty *et al.*, 2009 ▸) was used to obtain the dictionary file for the 2-hydroxyatrazine moiety; the guanine and atrazine compounds already had appropriate dictionary files available.

In order to remove the bound guanine and allow atrazine (2-hydroxyatrazine) to bind, the HEPES-based buffer in which the protein was formulated (50 m*M* HEPES pH 7.5 with 200 m*M* NaCl) with 5 m*M* atrazine added (from a 100 m*M* atrazine stock in DMSO) was added to the protein and allowed to rest for 1 h at room temperature before concentration in a spin concentrator. This process was repeated five times before the protein was concentrated to 16.9 mg ml^−1^ and drops were set up with microseeding in the same crystallization conditions as used for the native protein (the drops consisted of 150 nl protein, 130 nl crystallant and 20 nl seed stock). Crystals were obtained and handled as described above.

### 2.3. Surface plasmon resonance (SPR)   

All SPR experiments were performed using a Biacore S200 (GE Healthcare) biosensor with a CM5 chip. AtzT was coupled to the chip surface at 298 K in HBS-P+ buffer [10 m*M* HEPES pH 7.4, 150 m*M* NaCl, 0.05%(*v*/*v*) Tween 20] after activation with a 1:1 mixture of NHS/EDC [*N*-hydroxy­succinimide/*N*-ethyl-*N*′-(3-diethylaminopropyl)carbo­diimide]. The protein was diluted to 15 µg ml^−1^ at pH 7 and injected to immobilization levels of ∼7000 RU (∼5 min, 10 µl min^−1^). The surface was then blocked with 1 *M* ethan­olamine pH 8.0. Prior to immobilization the AtzT protein is bound to guanine (as demonstrated by crystallography and mass spectrometry); however, the SPR data reveal that the majority of the guanine dissociates in as little as 20 s (Supplementary Fig. S4). The relatively long time between protein immobilization and the first analyte injection (≥1 h) thus provides a surface that is free from guanine. All SPR binding experiments were performed at 293 K in the above immobilization buffer but with the addition of 2%(*v*/*v*) DMSO. Analytes were serially diluted (twofold) in SPR binding buffer, injected for 30 s contact time at 60 µl min^−1^ and then allowed to dissociate back to baseline. Each analyte titration was performed in triplicate. Binding sensorgrams were processed, solvent-corrected and double-referenced using *Scrubber* (BioLogic Software, Australia). To determine the binding affinities, responses at equilibrium for each analyte were fitted to a 1:1 steady-state affinity model. Where the top concentration (typically 200 µ*M*) for a compound was less than twofold above its *K*_D_, the responses were fitted using a global *R*_max_ determined with guanine (with sensorgrams scaled to the molecular weight). Adjusting the response by molecular weight is a potential source of error, although this is generally no more than twofold (Davis & Wilson, 2000 ▸). The compounds that were fitted to a local *R*_max_ (ammeline, guanine and adenine) all displayed equivalent *R*_max_ values (when responses were scaled to molecular weight); however, the potential that the other globally fitted compounds possess deviant refractive-index increments is a relevant caveat when interpreting compound affinities. The compounds melamine and ammelide appeared to contain trace amounts of the tightly binding ammeline (based on the heterogeneous curve shape and the observed off-rates); although a large discrepancy in affinity between these compounds was reliably demonstrated, an exact *K*_D_ value was not determined. Instead, a range is given for melamine and ammelide (*K*_D_ > 400 µ*M* and *K*_D_ > 100 µ*M*, respectively) to avoid overinterpretation. Sensorgrams and binding isotherms are displayed in Supplementary Fig. S4.

### 2.4. Mass spectrometry   

The accurate mass of recombinant proteins was confirmed by LC-ESI-MS. Samples were spiked with formic acid to a final concentration of 0.1%(*v*/*v*) and separated by reverse-phase liquid chromatography using a linear gradient from 0 to 80% mobile phase *B* [Dionex UltiMate 3000 RSLCnano System, Thermo Scientific; mobile phase *A*, 0.1%(*v*/*v*) formic acid; mobile phase *B*, 90%(*v*/*v*) acetonitrile/0.1%(*v*/*v*) formic acid] and ionized using an Apollo II electrospray source (Bruker) with a nebuliser pressure of 1 bar and a drying gas flow of 8 l min^−1^ at 473 K. A maXis II mass spectrometer (Bruker) was calibrated in positive-ion mode using ESI-TOF Low Concentration Tuning Mix (Agilent) and high-resolution MS raw data were processed with Bruker *Compass Data­Analysis* v.4.3.

To identify the unknown compound which was seen in AtzT by X-ray crystallography, we tested the original protein sample for the presence of an ∼150 Da small molecule (estimate based on the electron density at 1.67 Å resolution). Using ESI-MS in direct infusion mode, we discovered a monoisotopic mass of 151.0772 Da that was exclusively present in the spectra of the AtzT sample but not in spectra obtained from the protein-free crystallization solution. We performed a molecular-weight search of the *E. coli* Metabolome Database (http://ecmdb.ca/). This identified guanine (monoisotopic mass = 151.0997) as the closest match. In order to validate this possible hit we also fragmented the corresponding MH^+^ precursor ion at collision energies of 10–40 eV and searched the resulting MSMS spectrum against the METLIN metabolite database (https://metlin.scripps.edu). This yielded a unique, confident MSMS spectrum match for guanine (METLIN score 15, Δp.p.m. = 15, MSMS tolerance = 0.01 Da, collision energy 40 eV).

## 3. Results   

**Figure 1:**
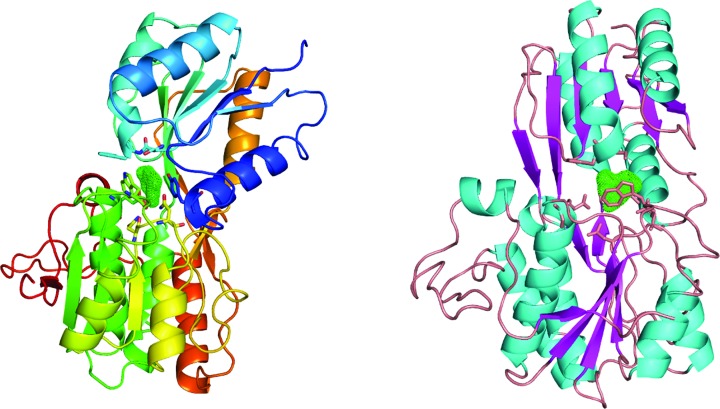
The image on the left is a cartoon representation coloured by Jones’ rainbow with the N-terminus in dark blue and the C-terminus in dark red. The green mesh represents the unknown difference density found after solving the protein structure sitting between the two domains. The image on the right is a cartoon representation that is coloured by secondary structure with α-helices in cyan and β-sheets in magenta. It is orientated with an approximate 90° rotation to the figure on the left.

**Figure 2:**
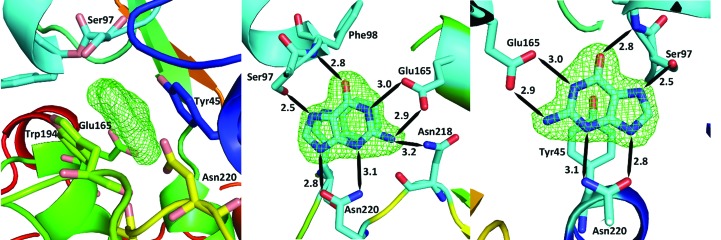
An enlarged image of the binding site between the two domains. The image on the left shows the difference density (*mF*_o_ − *DF*_c_, 3σ) before guanine was placed into the model. The relevant amino acids in the binding site are shown and the backbone is coloured by Jones’ rainbow as in Fig. 1 ▸(*a*). The middle and right figures are rotated plus and minus approximately 90° from the figure on the left and are coloured by atom type, with C in cyan, O in red and N in dark blue. Potential hydrogen bonds are represented as black lines; approximate distances between potential hydrogen-bond acceptors and donors are shown in Å and the binding residues are labelled. Note that the Asn218 is out of plane to the guanine, so the hydrogen on the Asn218 N atom is likely to interact with the π-cloud perpendicular to the plane of the atoms.

As periplasmic binding proteins often contain a twin-arginine translocation (TAT) signal at the N-terminus to target the protein to the periplasm (Palmer & Berks, 2012 ▸), the TAT signal sequence was identified using *TatP* (http://www.cbs.dtu.dk/services/TatP/) and *Protter* (http://wlab.ethz.ch/protter/start/). Primers were designed to amplify the gene sequence without the sequence encoding the TAT signal in order to facilitate heterologous expression. The X-ray crystallographic analysis shows that AtzT is a two-domain protein, with each domain containing a central β-sheet of 6–7 strands, with 2–3 α-helices on either side of this central β-sheet and a C-terminal extension of about 45 residues (see Fig. 1 ▸). The N-terminal domain is made up of residues 27–133 plus an extension from the C-terminal domain of residues 257–287, which forms a single α-helix and a β-strand that is on one edge of the β-sheet through the middle of the first domain. The second (C-terminal) domain runs from residue 134 to residue 315, minus the extension 257–287, and then has an extension from 316 to 360 which adopts a predominantly random-coil structure. Between the two domains we find the binding site, in which clear density (>10σ) was seen for an unknown compound in both the original 1.87 Å resolution SAD structure and the subsequent 1.67 Å resolution native structure. Mass-spectrometric analysis allowed us to determine that the compound had a mass of 151 Da, and fragmentation analysis of this compound led us to believe that the compound was guanine. Guanine was placed into the electron density, where it refined well, and the resulting hydrogen-bond pattern to the binding-site residues satisfied every N and O atom of guanine. As can be seen in Table 1 ▸, the average *B* factors for the guanine are comparable to the average *B* factors of the protein, and this is also true of the *B* factors of the binding-site residues. The binding site consists of the Asn218, Asn220, Glu165 and Ser97 side chains, which make hydrogen bonds, and the Phe98 backbone N atom, which makes a hydrogen bond to the carbonyl O atom of guanine (see Fig. 2 ▸). The guanine ring is packed between (sandwiched by) Tyr45 and Trp194. Every possible heteroatom of the guanine appears to engage with the protein. We note that Asn218 is in an unconventional rotamer [180° rotation to rotamer 3 (**t**-20°) in *Coot*] and that the interaction with the guanine amine is out of plane with the atoms of this compound. Flipping the Asn218 side chain to have the O atom of the side chain presented to the guanine would require a hydrogen of the amine to move out of plane at an ∼65° angle to generate a hydrogen bond. As neither of these scenarios seemed to be likely, we decided to model the Asn218 in the unconventional rotamer state, which allows a hydrogen of the side chain to interact with the π-cloud of guanine. Additionally, the Asn218 forms a hydrogen-bond network with Asn169, which is oriented within hydrogen-bonding distance of the Glu165 backbone carbonyl. Asn169 is also positioned to hydrogen-bond to the O atom of the Thr139 side chain.

The structure of AtzT allows us to classify this protein into cluster B of the solute-binding proteins (Berntsson *et al.*, 2010 ▸). Cluster B has three connecting strands between the two domains and there are no α-helices interrupting these connections, placing AtzT in the B-I cluster. According to *PDBeFold*, AtzT is most similar to the entry with PDB code 3s99 (Seattle Structural Genomics Center for Infectious Disease, unpublished work), which is also classified as a B-I cluster protein and had electron density in the binding site for a purine. *Post hoc* analysis of the structure and sequence showed that PDB entry 3s99 has 54% sequence identity and an r.m.s.d. of 1.2 Å (over ∼330 residues) to AtzT. Using *Phaser* with PDB entry 3s99 showed that the AtzT structure could have been solved by molecular replacement using this model. PDB entry 3s99 is annotated as a ‘basic membrane lipoprotein from *Brucella melitensis*’, although the deposited model has an adenine moiety modelled into the binding site, suggesting that it is another purine-binding SBP.

As AtzT was thought to be a protein that bound atrazine, we used surface plasmon resonance (SPR) to test the binding activity of the protein to several different nucleobases and to atrazine and atrazine metabolites and analogues (see Table 2 ▸). The shape of the SPR curve for atrazine suggested a heterogeneous analyte; liquid-chromatography and mass-spectrometric analysis of the atrazine starting material showed that 5% of the starting material was 2-hydroxyatrazine (data not shown). Fresh atrazine and 2-hydroxyatrazine were reordered and tested, and clean sensorgrams were obtained for each (see Supplementary Fig. S4), with *K*_D_ values of ∼1.5 m*M* and 2.2 µ*M*, respectively. 2-Hydroxyatrazine has a similar affinity as adenine (*K*_D_ ≃ 2.5 µ*M*) but binds more weakly than guanine (*K*_D_ ≃ 110 n*M*). The other nucleobases display limited binding (*K*_D_ ≥ 620 µ*M*). Guanine is the tightest-binding compound tested, with a *K*_D_ of ∼110 n*M*. It is interesting to note that ammeline and ammelide bind very differently to AtzT, ammeline with a *K*_D_ of 360 n*M* and ammelide with a *K*_D_ of >100 µ*M* (>250-fold difference), despite these compounds differing by a single nitrogen-to-oxygen substitution on the same triazine ring. Similarly, substituting the O atom of ammeline with an N atom (*i.e.* melamine; *K*_D_ > 400 µ*M*) also reduces binding.

**Figure 3:**
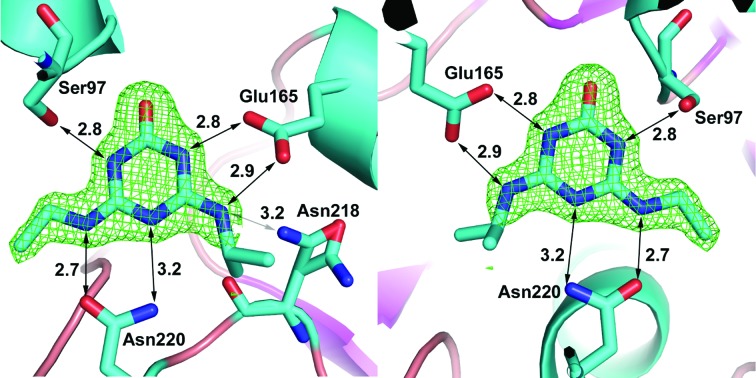
An enlarged image of 2-hydroxyatrazine in the binding site. The difference map (*mF*_o_ − *DF*_c_) is set to 3σ and the compound has good density, with the exception of the isopropyl group, which can be found in more than one orientation but has clashes with the protein (for example Asn218) in all possible orientations. The same residues that make hydrogen bonds to guanine make very similar interactions with the 2-hydroxyatrazine heteroatoms and the compound is sandwiched between Tyr45 and Trp194 in a similar fashion to guanine. C atoms are in cyan, O atoms are in red and N atoms are in dark blue.

The structure with 2-hydroxyatrazine diffracted to a nominal resolution of 1.65 Å; it belongs to the same space group (*P*2_1_) and has the same fold and closed form as the other structures that we have determined. This structure was obtained prior to the SPR data and used the aforementioned 2-hydroxyatrazine-contaminated sample of atrazine for co-crystallization. Modelling atrazine into the structure resulted in distinct negative difference density at the Cl atom, which was alleviated completely upon replacement with 2-hydroxy­atrazine. The occupancy of the binding site is thus suggested to be effectively entirely 2-hydroxyatrazine, which agrees with the affinity data presented. The crystallography results thus verify the SPR results: a contaminant found in low abundance (about 5% of the total compound sample, but ∼700-fold more tightly binding) is the entity that is found bound in the structure. The same residues contribute to the binding of 2-hydroxyatrazine as to the binding of guanine in the binding site: Asn220, Glu165 and Ser97, with the ring packed on either side by Tyr45 and Trp194. 2-Hydroxyatrazine does not fit as well into the binding site as guanine and has some steric clashes on the side with the isopropyl moiety (see Fig. 3 ▸). It has well defined density except around the isopropyl moiety, which shows close contacts with Asn218 (2.4–3.2 Å depending on the atom and the chain) and with Asn220 (3.0–3.2 Å to the N atom of the side chain). The Asn218 residue has weaker density and can be found in multiple conformations. The ethyl moiety on the other side fits the density much better and generally does not make extensive clashes with the protein residues (3.1–3.2 Å from the O atom of the Asn220 side chain). The N atoms of 2-hydroxyatrazine are well within hydrogen-bonding distances of Asn220, Glu165 and Ser97 and the hydroxyl moiety makes a bond (2.9 Å on average) to the backbone N atom of Phe98, analogous to the carbonyl of guanine.

## 4. Discussion   

We have expressed, purified, crystallized and determined the structure of AtzT using SAD phasing from an ethylmercury derivative. We found strong density in the binding site of AtzT that represented a small aromatic compound and have determined by mass spectrometry that this small molecule is very likely to be guanine. Additionally, we have run SPR against a bank of small-molecule compounds of similar size and guanine is the tightest-binding compound tested, binding with a *K*_D_ of around 110 n*M*. We have also determined the structure of AtzT bound to 2-hydroxyatrazine and shown that the binding affinity of this substrate for AtzT is 2.2 µ*M*, which is significantly (>600 fold) tighter than atrazine at ∼1.5 m*M*.

The *Pseudomonas* sp. strain ADP genome contains 112 genes annotated as ‘substrate-binding proteins’ (Supplementary Table S1), none of which have similarity to AtzT. It also contains genes that code for enzymes for *de novo* purine synthesis (KSW22506.1, KSW23190.1, KSW23286.1, KSW23824.1, KSW23941.1, KSW24282.1, KSW25158.1, KSW25646.1 and KSW25647.1). Additionally, a purine permease (KSW22651.1) was identified on the genome, suggesting that *Pseudomonas* sp. strain ADP can also take up purine *via* at least one other pathway. The *atzT* gene is found with the other *atz* genes on the 97 kb plasmid pADP-1 in *Pseudomonas* sp. strain ADP. To date, no obvious gene involved in either chemotaxis or transport had been identified on the pADP-1 plasmid. Our finding suggests that *atzT* codes for a periplasmic transport protein. It has a TAT signal at the N-terminus and has sequence and structural homology to many other periplasmic binding proteins. Sequence comparison of AtzT with other proteins found in UniProt gives a high number of hits for proteins that are classified as ‘nucleoside-binding protein’ or ‘purine-binding protein’ from such organisms as *Massilia* (84% identity; A0A1G6IR71), *Rugamonas rubra* (76% identity; A0A1I4KBL4_9GAMM) and *Duganella* (71–76% identity; A0A1E7WQA5_9BURK). Atrazine is a modified *s*-triazine compound that has some similarity to purines, so it seems reasonable to hypothesize that AtzT is a purine-binding protein that can promiscuously bind atrazine and atrazine derivatives. After identifying the unknown compound binding to AtzT as guanine (using a combination of X-ray crystallography, mass-spectrometric analysis and SPR) and searching sequence databases for the sequences of homologous proteins, we believe that AtzT is a purine-binding protein that can accept 2-hydroxyatrazine (and possibly atrazine) from the environment when it is present and guanine is absent.

We speculate that the promiscuous uptake of 2-hydroxy­atrazine (which is a likely contaminant in most atrazine samples) could prove to be an evolutionary benefit for those organisms living in some of the more intensively farmed lands where atrazine has been used in abundance as a herbicide (Europe prior to 2004, and the US and Australia currently, as well as Asia). Organisms such as *Pseudomonas* sp. strain ADP have been shown to survive on atrazine as a nitrogen/carbon source and have evolved the necessary pathways to do so effectively (Govantes *et al.*, 2009 ▸). Several other organisms have been shown to possess similar pathways for the use of atrazine (and other triazine-based products) as nutrients/energy sources (Mongodin *et al.*, 2006 ▸; Young *et al.*, 2006 ▸; Kapley *et al.*, 2013 ▸; Nakamoto *et al.*, 2016 ▸; Devers-Lamrani *et al.*, 2016 ▸).

Periplasmic binding proteins have been engineered to serve as biosensors, exploiting the large conformation change induced by ligand binding to affect changes in fluorescence resonance energy transfer (FRET) and bio­luminescence resonance energy transfer (BRET) signals from fused fluorescent and luminescent tags (Ye & Schultz, 2003 ▸; Dacres *et al.*, 2013 ▸). The high-resolution structures presented here provide a depth of information that will allow the re-engineering of AtzT for greater specificity for contaminants such as atrazine and melamine, and potentially for medically relevant compounds such as barbituric acid or related barbiturates.

## Supplementary Material

PDB reference: AtzT, ethylmercury phosphate derivative, 6pi5

PDB reference: native (guanine-bound), 6pi6

PDB reference: complex with 2-hydroxyatrazine, 6pii

Supplementary Figures and Table. DOI: 10.1107/S2059798319013883/jb5014sup1.pdf

## Figures and Tables

**Table 1:** X-ray data statistics Values in parentheses are for the highest resolution bin.

Ligand	Ethylmercury phosphate	Guanine	2-Hydroxyatrazine
PDB code	6pii	6pi5	6pi6
Data collection			
Space group	*P*2_1_	*P*2_1_	*P*2_1_
*a*, *b*, *c* (Å)	98.9, 63.1, 120.4	99.0, 63.1, 120.4	98.8, 62.8, 120.2
α, β, γ (°)	90, 93.5, 90	90, 93.6, 90	90, 93.4, 90
Wavelength (Å)	1.0082	0.9537	0.9537
Resolution (Å)	49.4–1.87 (1.90–1.87)	49.3–1.67 (1.70–1.67)	49.0–1.65 (1.68–1.65)
Completeness (%)	97.8 (80.1)	99.3 (93.2)	100.0 (100.0)
*R*_merge_ (%)	0.140 (0.669)	0.083 (0.643)	0.086 (1.265)
*R*_p.i.m._ (%)	0.032 (0.246)	0.034 (0.285)	0.035 (0.515)
Mean *I*/σ(*I*)	13.5 (3.1)	11.9 (2.7)	10.8 (1.5)
No. of unique reflections	119947 (4833)	171863 (7958)	176707 (8668)
Multiplicity	19.6 (7.4)	6.8 (5.8)	6.8 (7.0)
CC_1/2_	0.998 (0.809)	0.998 (0.769)	0.998 (0.653)
Anomalous completeness (%)	97.1 (70.5)		
Anomalous multiplicity	9.8 (4.1)		
No. of Hg atoms	4		
Refinement			
Resolution (Å)	49.4–1.87 (1.92–1.87)	49.3–1.67 (1.71–1.67)	49.0–1.65 (1.69–1.65)
No. of reflections	114140 (7356)	163254 (11606)	167790 (12348)
*R*_work_ (%)	15.5 (22.8)	15.6 (22.9)	16.2 (28.1)
*R*_free_ (%)	18.3 (27.4)	18.4 (26.1)	18.2 (30.7)
Total No. of atoms	10867	11222	11186
No. of ligand atoms	44	44	112
No. of waters	648	917	798
Wilson *B* value (Å^2^)	32.7	24.4	27.5
Mean *B* value (Å^2^)			
Protein	34.7	25.5	28.8
Waters	39.3	33.6	36.9
Ligand	37.1	23.6	24.4
R.m.s.d., bond lengths (Å)	0.011	0.012	0.012
R.m.s.d., bond angles (°)	1.544	1.650	1.602
Ramachandran analysis (%)			
Preferred	97.7	98.1	97.8
Allowed	2.3	1.9	2.2
Outliers	0	0	0

**Table 2:** SPR kinetic values

Compound	Chemical structure	*K*_D_ † (µ*M*)
Atrazine	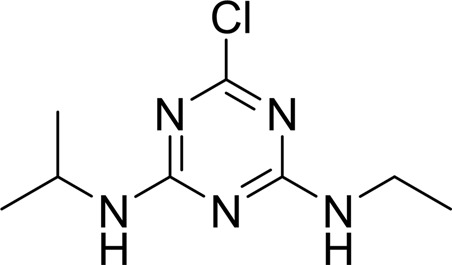	1500 ± 200
2-Hydroxyatrazine	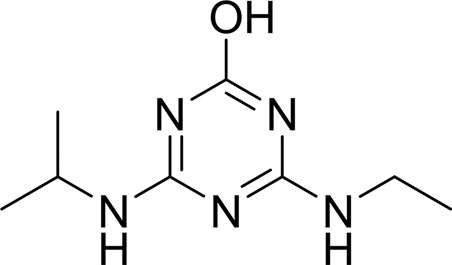	2.2 ± 0.2
Guanine	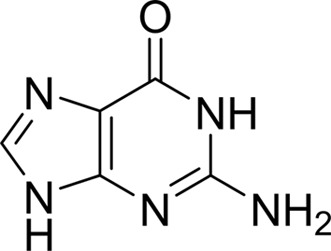	0.11 ± 0.02
Ammeline	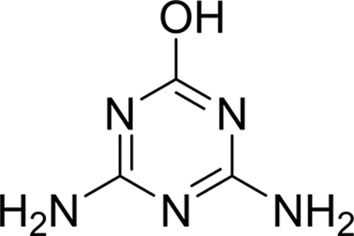	0.36 ± 0.02
Adenine	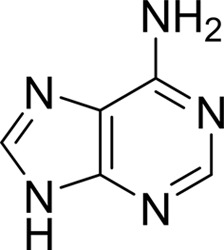	2.5 ± 0.3
Cyanuric acid	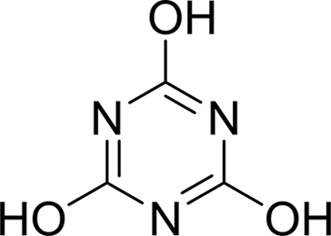	340 ± 30
Thymine	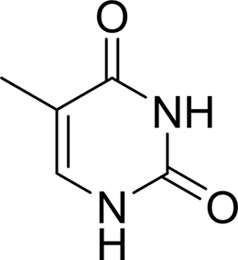	620 ± 40
Cytosine	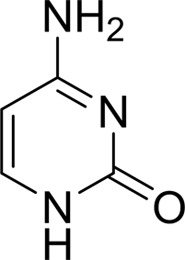	800 ± 90
Uracil	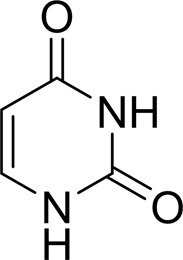	1600 ± 300
Ammelide	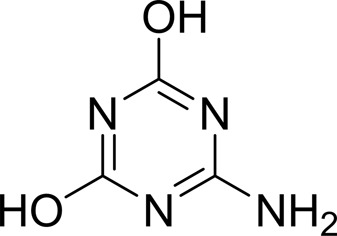	>100‡
Melamine	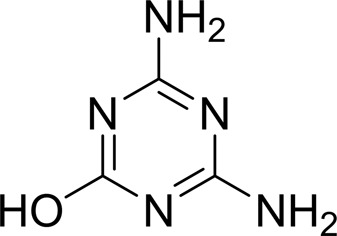	>400‡
4-Iodophenylboronic acid	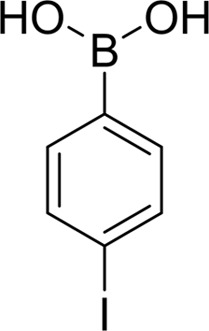	5000 ± 1000
Barbaturic acid	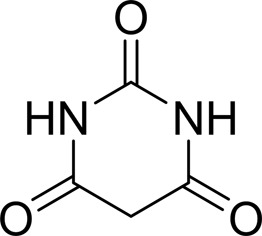	>8000
Barbitone	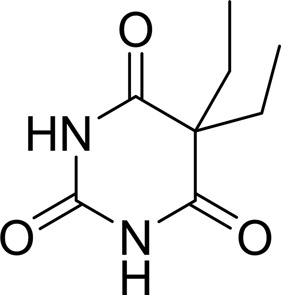	>8000
Tetrabromoterephthalic acid	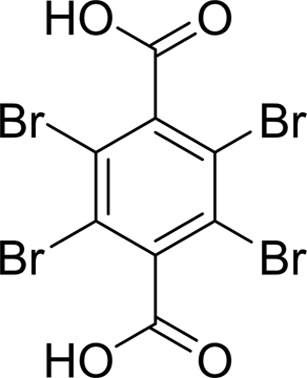	>8000
GTP	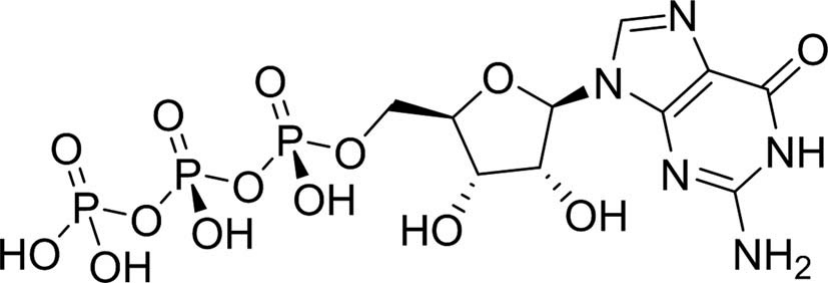	>8000

† Mean ± standard deviation (*n* = 3).

‡ The sample contains trace contamination with ammeline.
