# Supplementary material for: The evolving story of AtzT, a periplasmic binding protein
Source: Acta Crystallogr D Struct Biol. 2019 Oct 31;75(Pt 11):995–1002. doi: 10.1107/S2059798319013883 (PMC6834077; doi:10.1107/S2059798319013883)
Supplement: Supplementary file 1 [file d-75-00995-sup1.pdf]

STRUCTURAL
BIOLOGY

Volume 75 (2019)

Supporting information for article:

The evolving story of AtzT, a periplasmic binding protein

Matthew L. Dennis, Lygie Esquirol, Tom Nebl, Janet Newman, Colin Scott and Thomas S. Peat

MGSSHHHHHHSSGLVPRGSHMQEPLKVAFVYAGPVSDAGYTYAHDQGRLAMEKNLGAKV
KSSYVENVPEGADAERVIRKLAADGNKLIFTTSFGFMNPTERVAKAFPNVVFEHATGVKLAK
NLGVYESRQYEGTYLQGVLAAKMTKTGVIGFVGSFPVPEVIRNINAYTLGAQSVNPKIKTKVI
WVSTWYDPAKERQAAETLIAQGADVLTQNTNSPATLQVAQEKGKYAFGCDADMSKFAPKA
HLTASISNWGDFYTKTAQAVMAGTWKSEEVHWGMAEGMVKMAPLNAAVPPDAAKLFEEK
KAAMVSGKIKPFQGPLKDQSGAVKVAAGSDLPLASLKGMNWYVQGVEGTIPK

Figure S1 Protein sequence of the AtzT from *Pseudomonas* sp. strain ADP, lacking the tat signal in N-terminal region. In red is shown the 6xHis-tag and the thrombin cleavage sites added through the cloning.

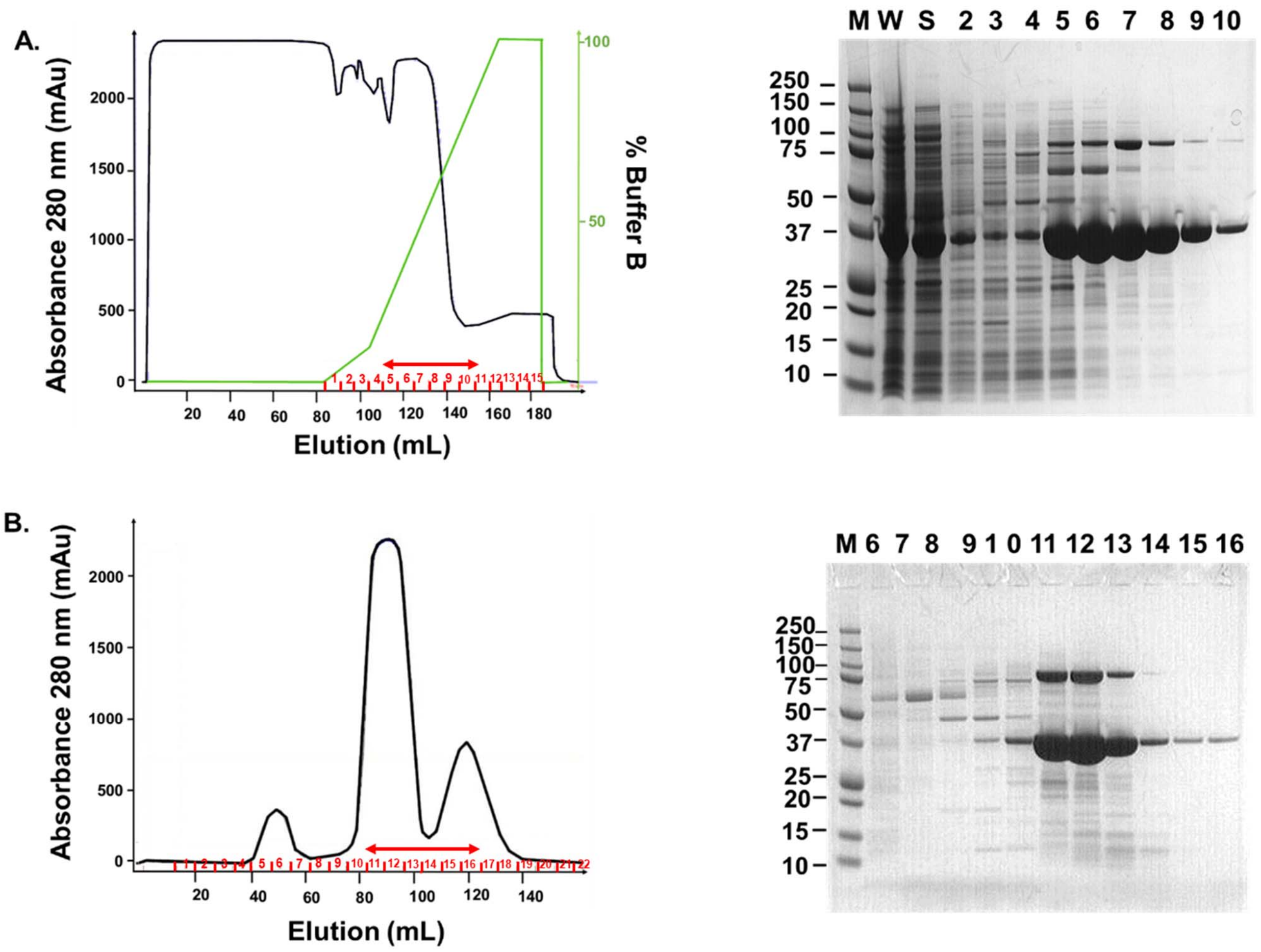

Figure S2 Purification of AtzT. A) NiNTA trace and SDS-PAGE analysis of the fractions 2–10, fractions 5-10 were pooled and are shown by the red arrow at the bottom of the trace, they were concentrated before being run on the S200 size exclusion column; B) Size exclusion trace and SDS-PAGE analysis of the fractions 6–16, fractions 11-16, shown by the red arrow at the bottom of the trace, were concentrated and used for further work; M: mw markers, W: whole cell extract; S: soluble extract, the expected size of AtzT is 38.7 kDa.

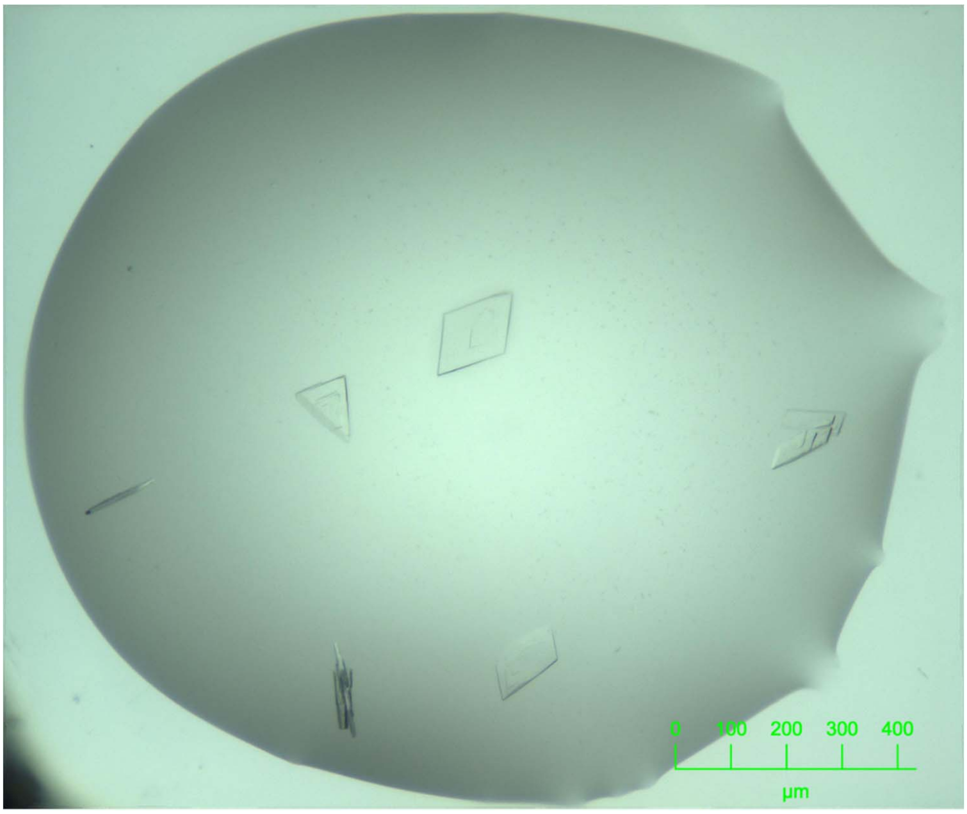

Figure S3 Crystals of AtzT grown in 7.39% (v/v) 2-methyl 2,4-pentanediol, 4.16% (v/v) polyethylene glycol 400, 1.15 M trisodium citrate. Drop consisted of 200 nL protein at 20 mg/mL, 180 nL reservoir solution and 20 nL of a seedstock. Crystal droplet was set up in SD-2 plates from Molecular Dimensions, UK, and was grown at 293 K.

Figure S4 SPR sensorgrams (left) and fit to a 1:1 steady-state model (right). χ^2 values are shown with respect to each replicate.

Guanine<br><br>χ^2 = 0.134 RU^2<br>χ^2 = 0.0865 RU^2<br>χ^2 = 0.137 RU^2	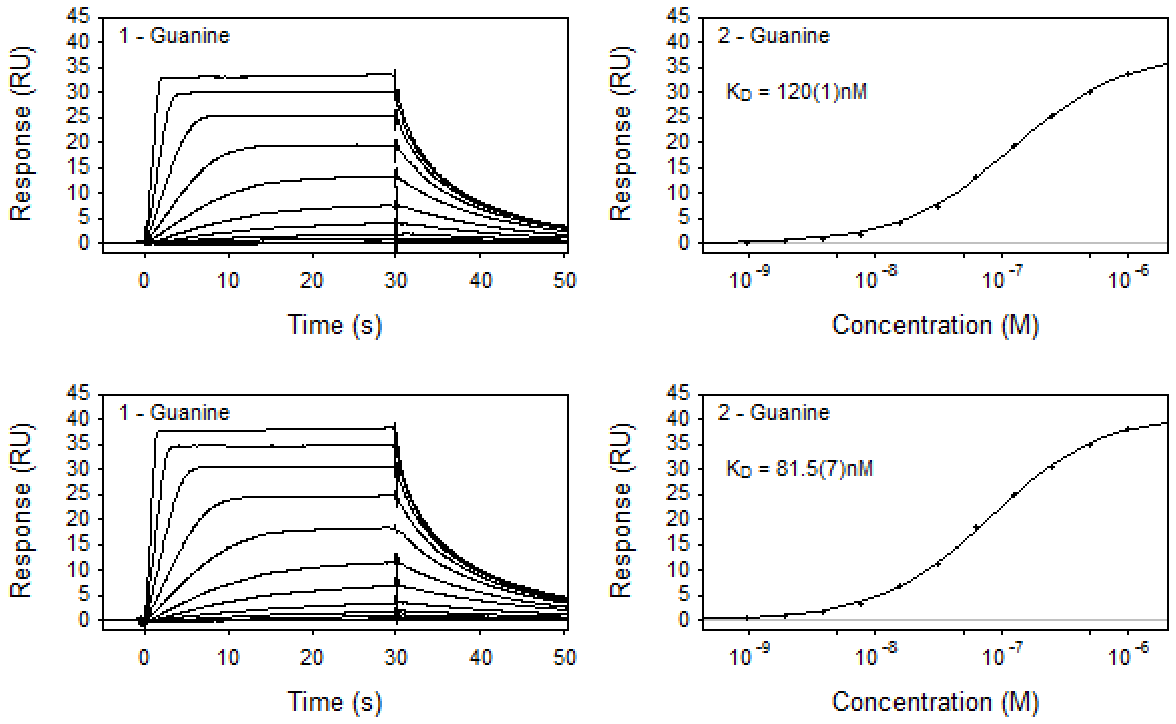

<table>
<tr>
<td></td>
<td>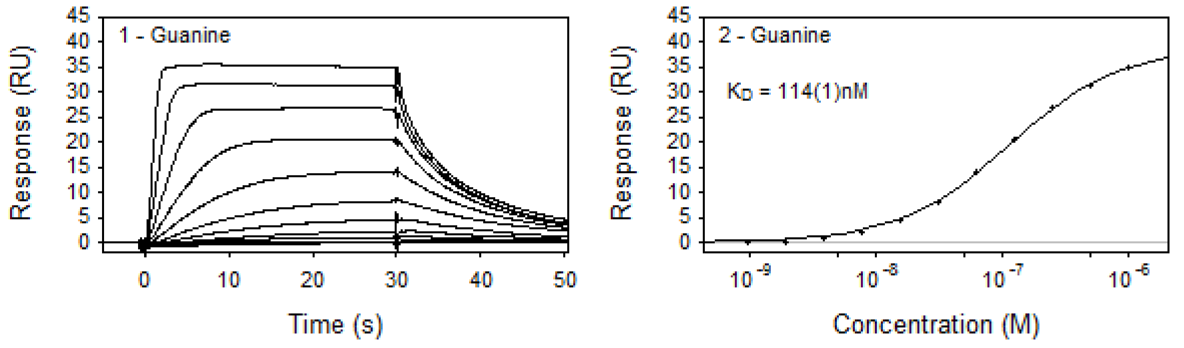
</td>
</tr>
<tr>
<td>Adenine

χ^2 = 0.0524 RU^2
χ^2 = 0.0116 RU^2
χ^2 = 0.0431 RU^2</td>
<td>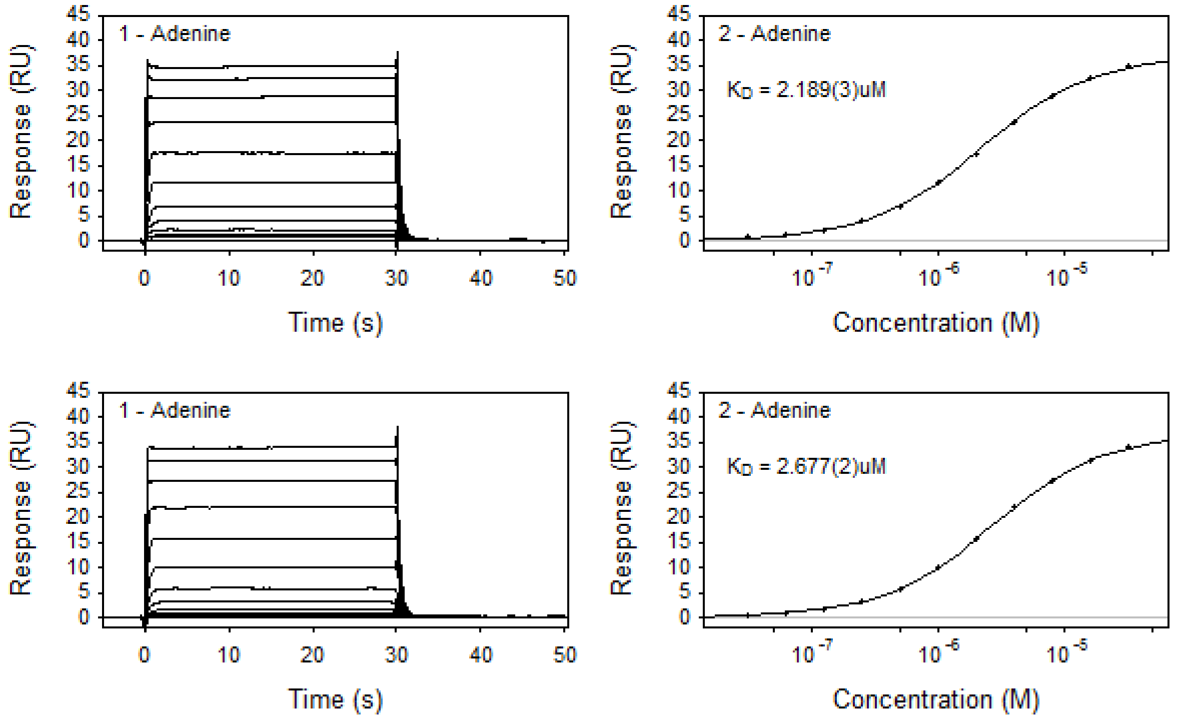
</td>
</tr>
</table>

<table>
<tr>
<td></td>
<td>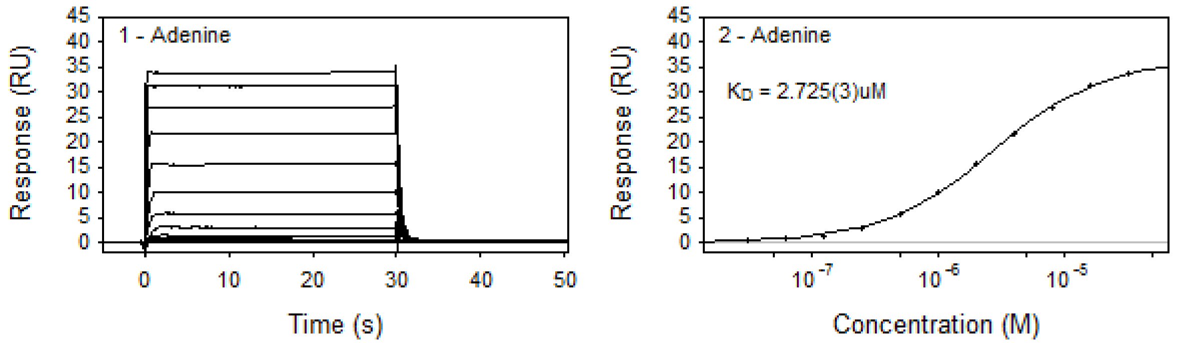
</td>
</tr>
<tr>
<td>Cytosine

χ^2 = 0.0033 RU^2
χ^2 = 0.0047 RU^2
χ^2 = 0.0839 RU^2</td>
<td>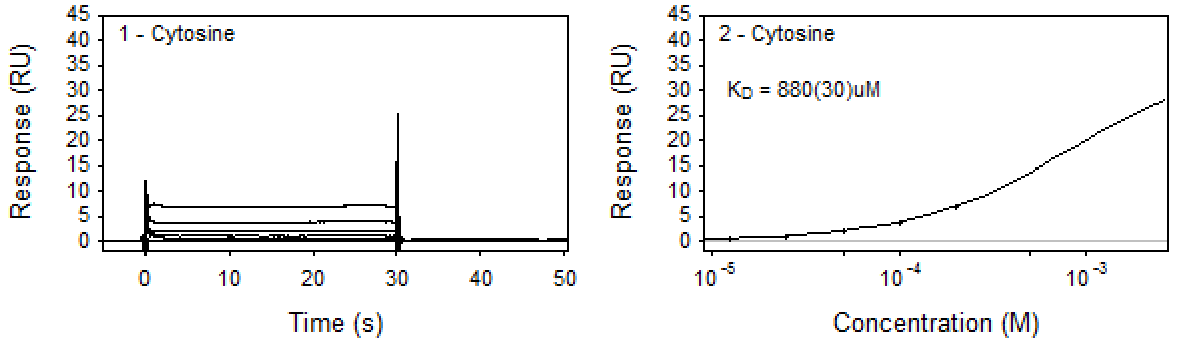

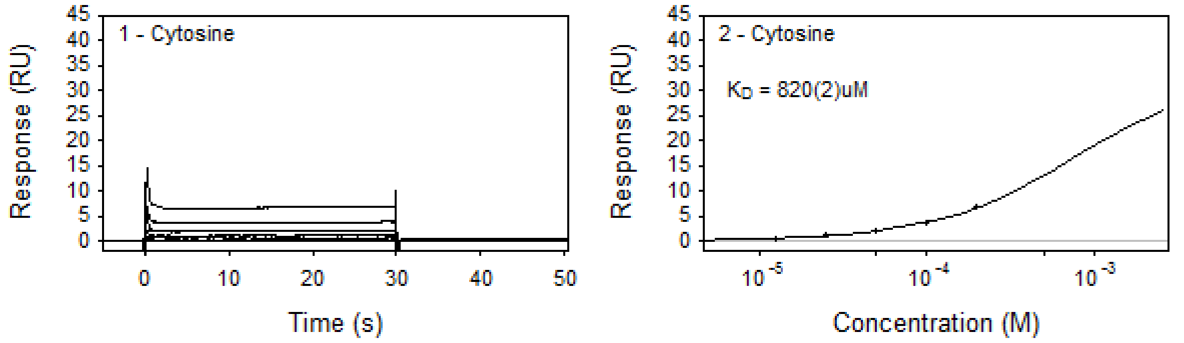
</td>
</tr>
</table>

	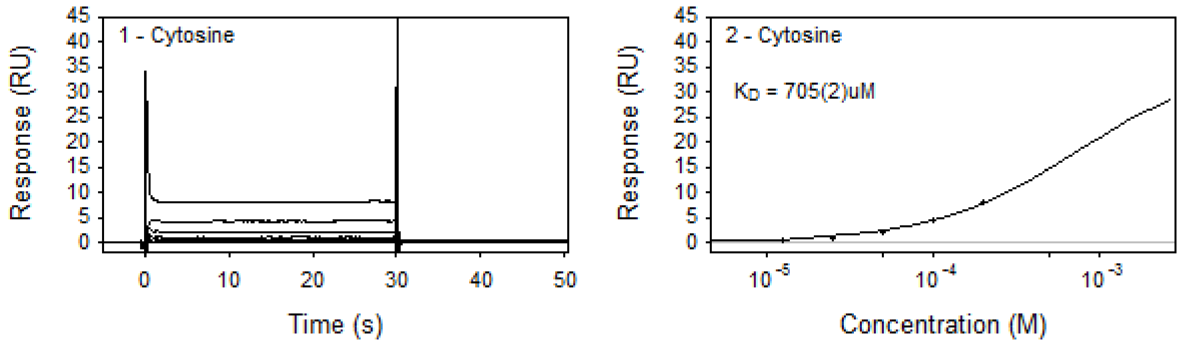
Thymine<br><br>χ^2 = 0.0313 RU^2<br>χ^2 = 0.0138 RU^2<br>χ^2 = 0.0127 RU^2	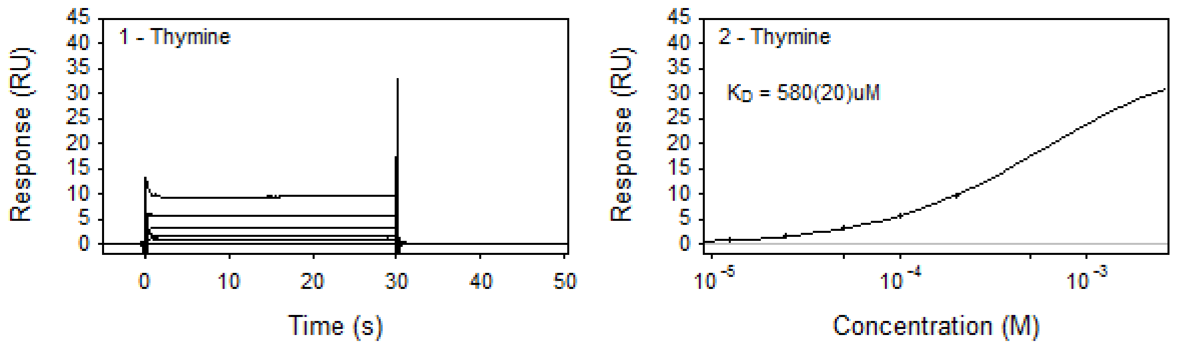

<table>
<tr>
<td></td>
<td>
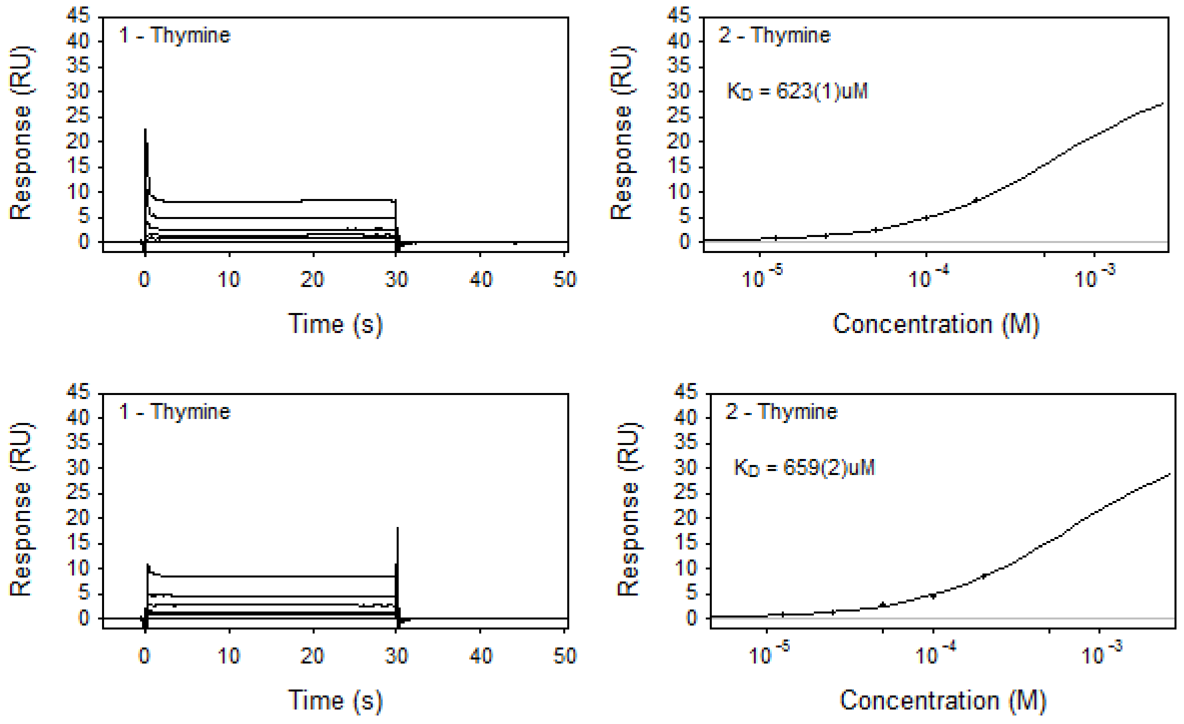

</td>
</tr>
<tr>
<td>Barbaturic acid

χ^2 = 0.0195 RU^2
χ^2 = 0.0290 RU^2
χ^2 = 0.0074 RU^2</td>
<td>
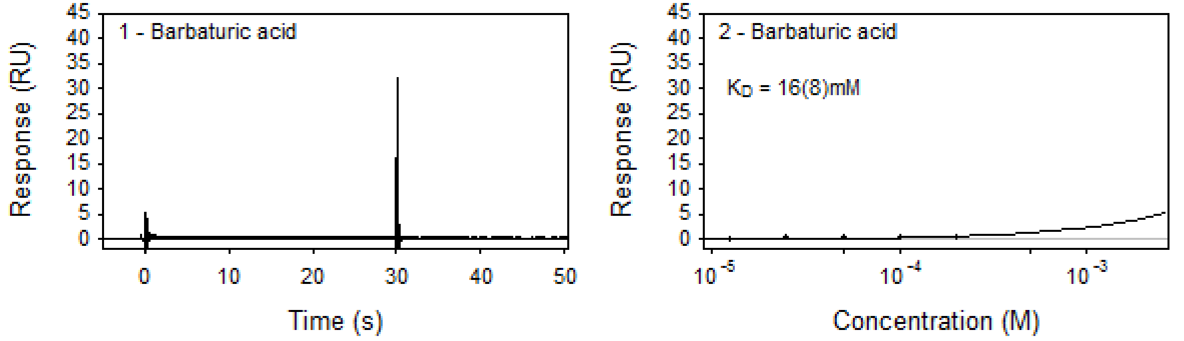

</td>
</tr>
</table>

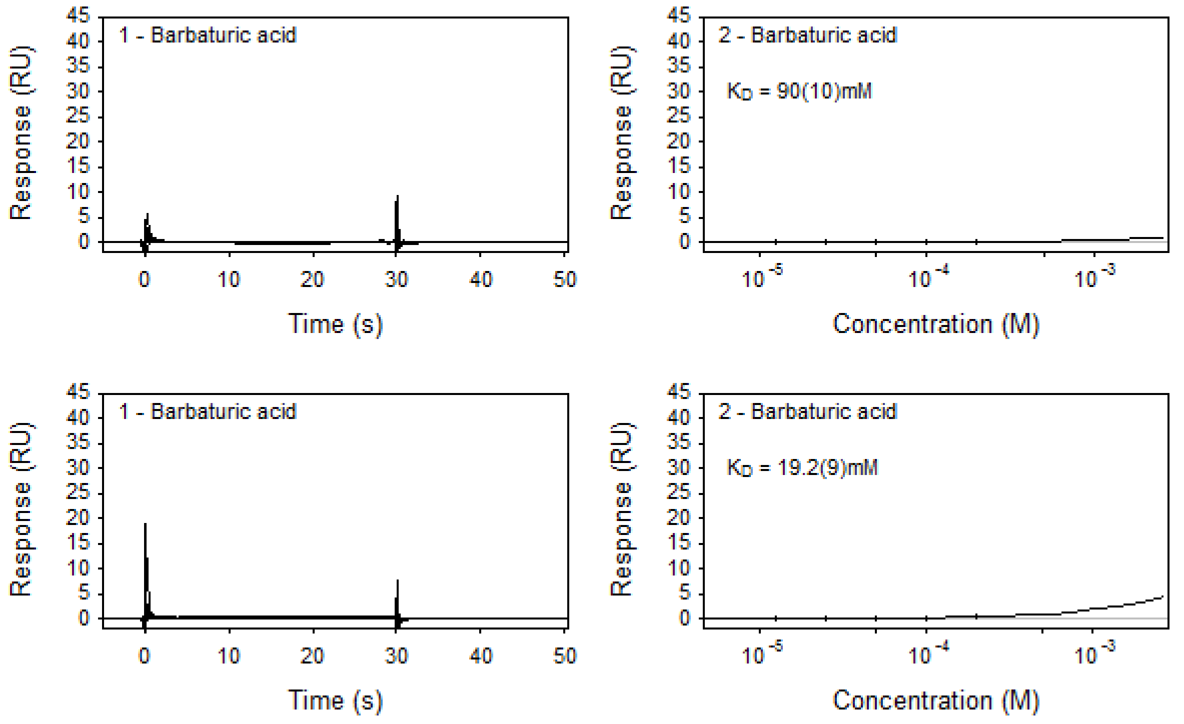

Atrazine

$\chi^2 = 0.0304$ RU^2

$\chi^2 = 0.0246$ RU^2

$\chi^2 = 0.0889$ RU^2

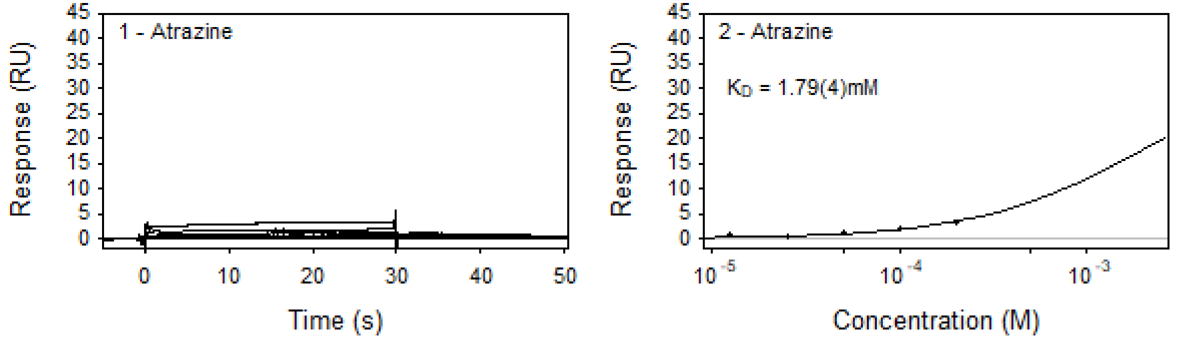

	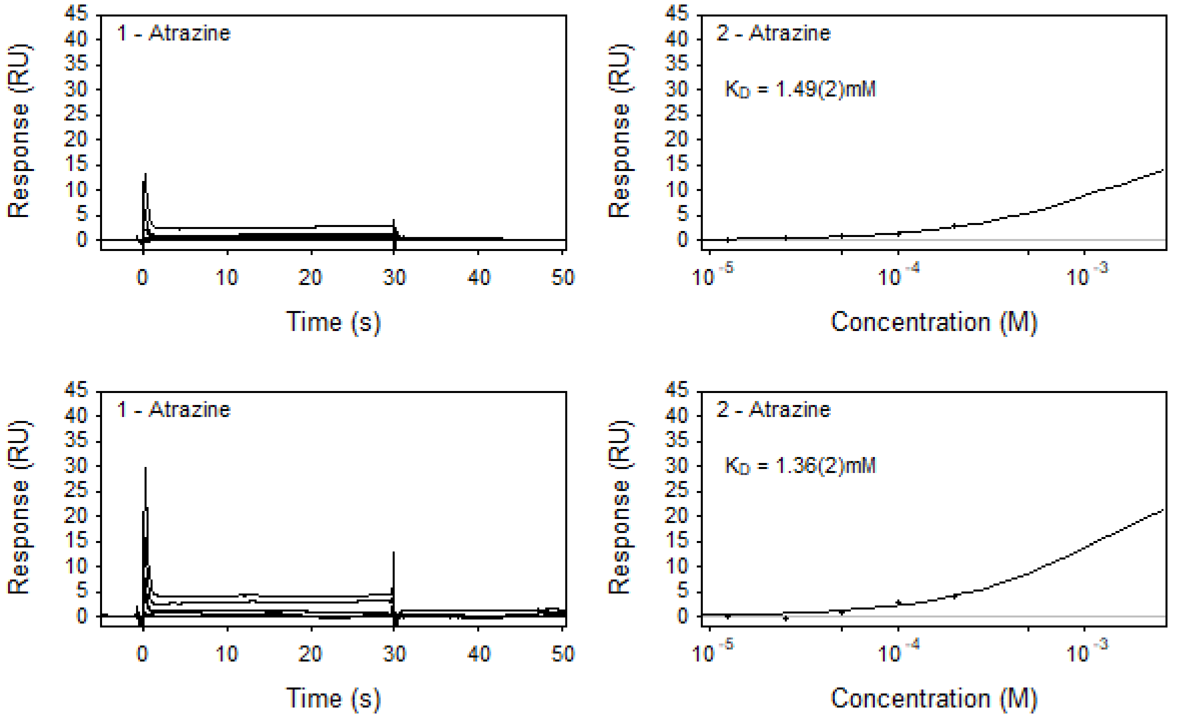
2-hydroxyatrazine<br><br>$\chi^2 = 0.0324\ RU^2$<br>$\chi^2 = 0.0242\ RU^2$<br>$\chi^2 = 0.182\ RU^2$	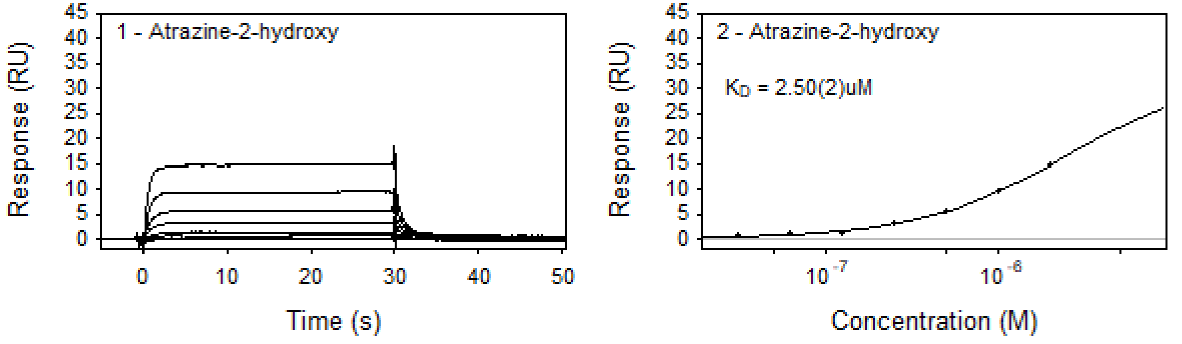

<table>
<tr>
<td></td>
<td>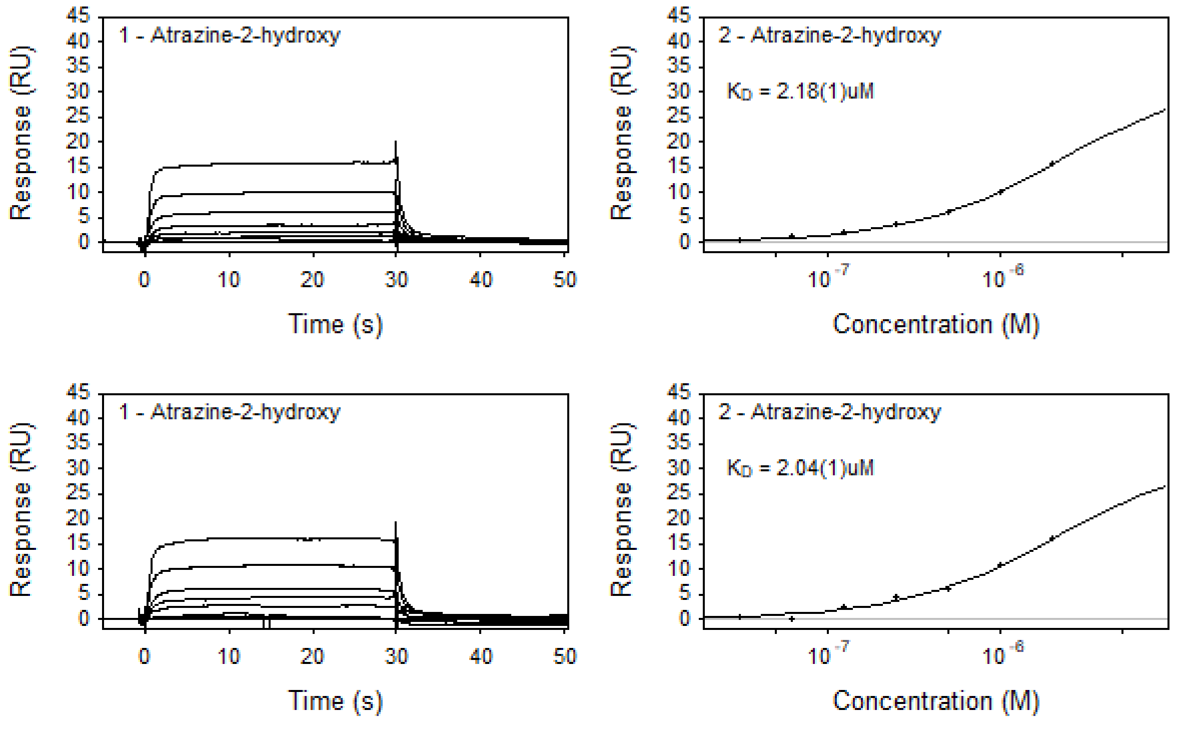
</td>
</tr>
<tr>
<td>Cyanuric acid

$\chi^2 = 0.271\ RU^2$
$\chi^2 = 0.0907\ RU^2$
$\chi^2 = 0.0536\ RU^2$</td>
<td>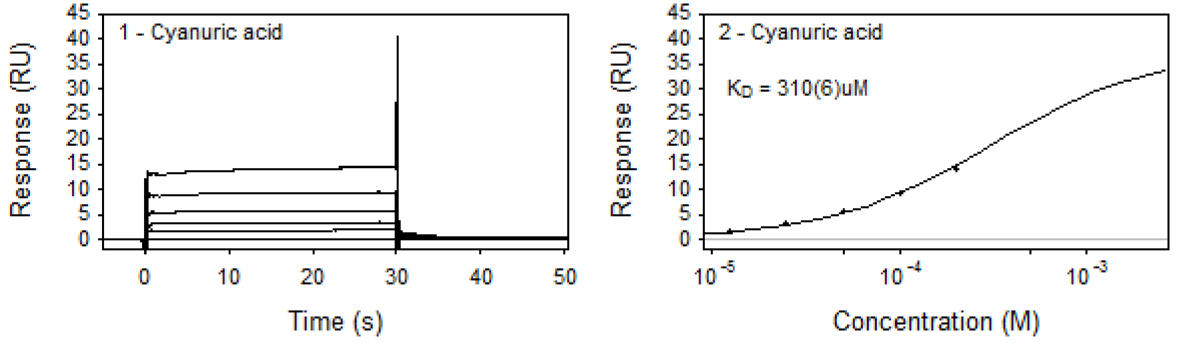
</td>
</tr>
</table>

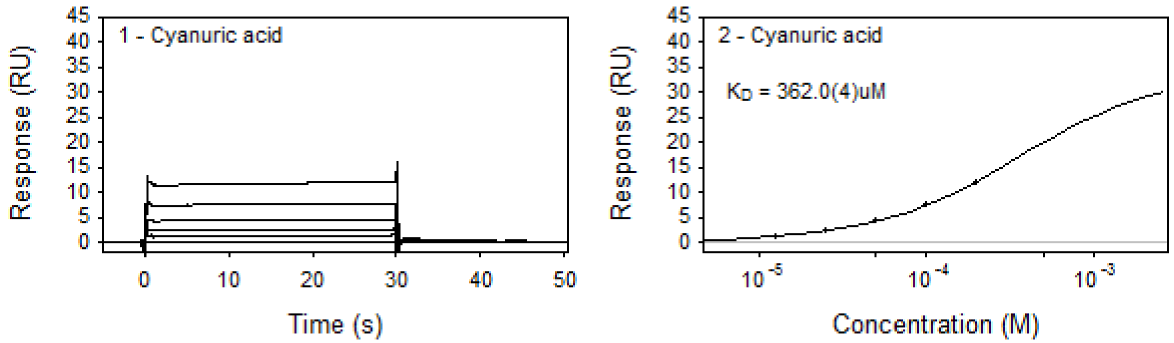

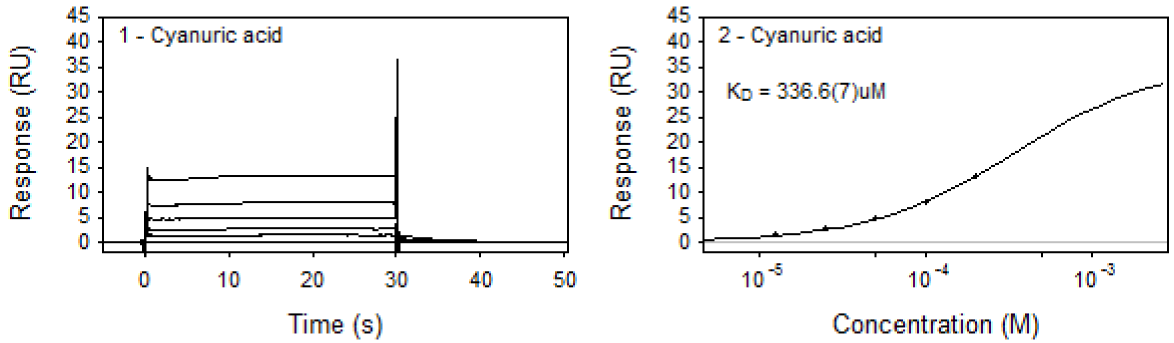

4-Iodophenylboronic acid

$\chi^2 = 0.0162\ RU^2$

$\chi^2 = 0.0009\ RU^2$

$\chi^2 = 0.0029\ RU^2$

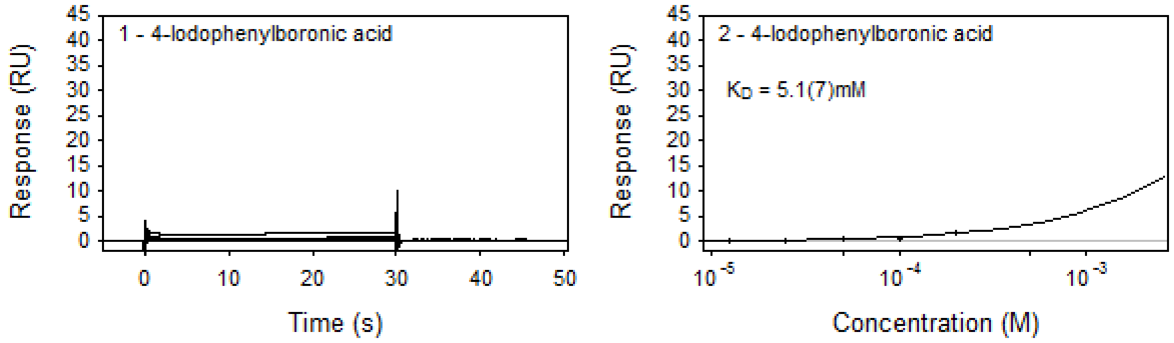

<table>
<tr>
<td></td>
<td>
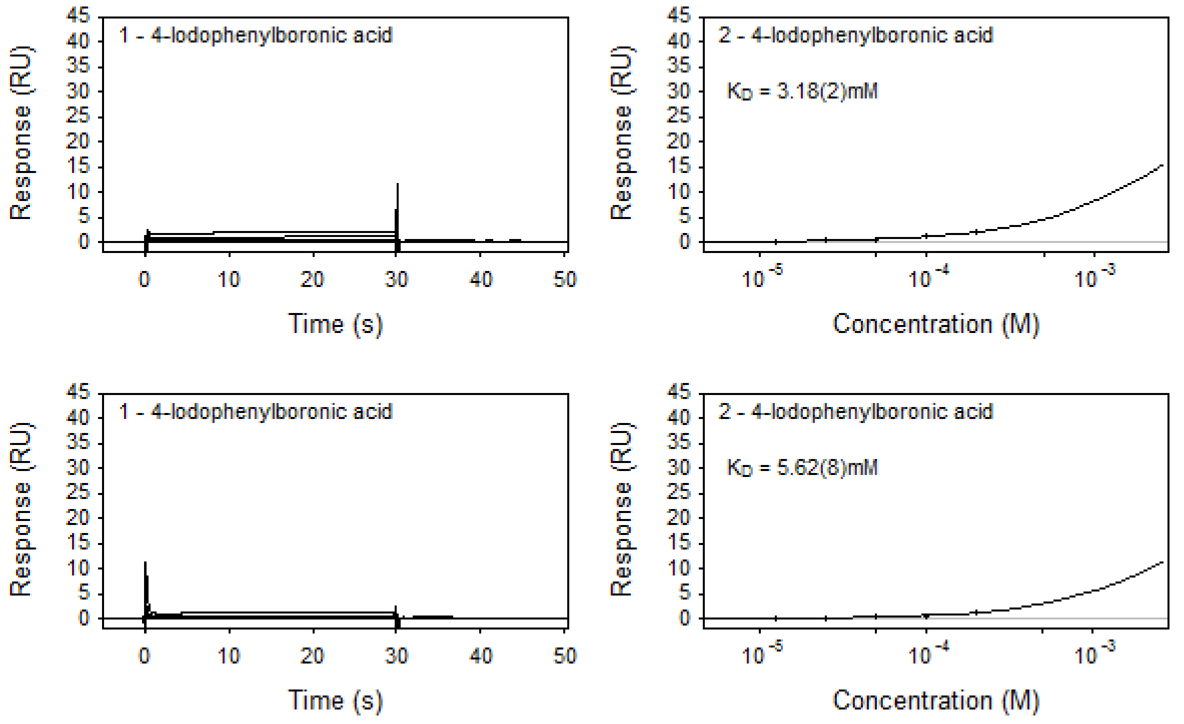

</td>
</tr>
<tr>
<td>Barbitone

χ^2 = 0.0059 RU^2
χ^2 = 0.0025 RU^2
χ^2 = 0.0029 RU^2</td>
<td>
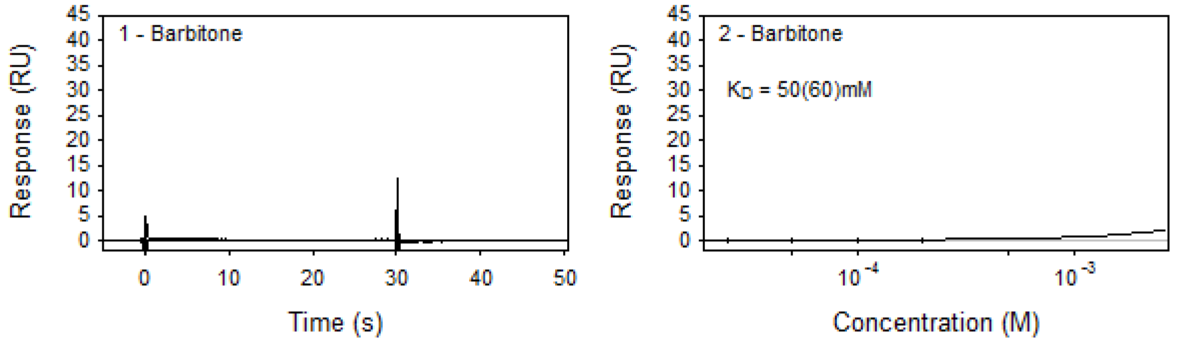

</td>
</tr>
</table>

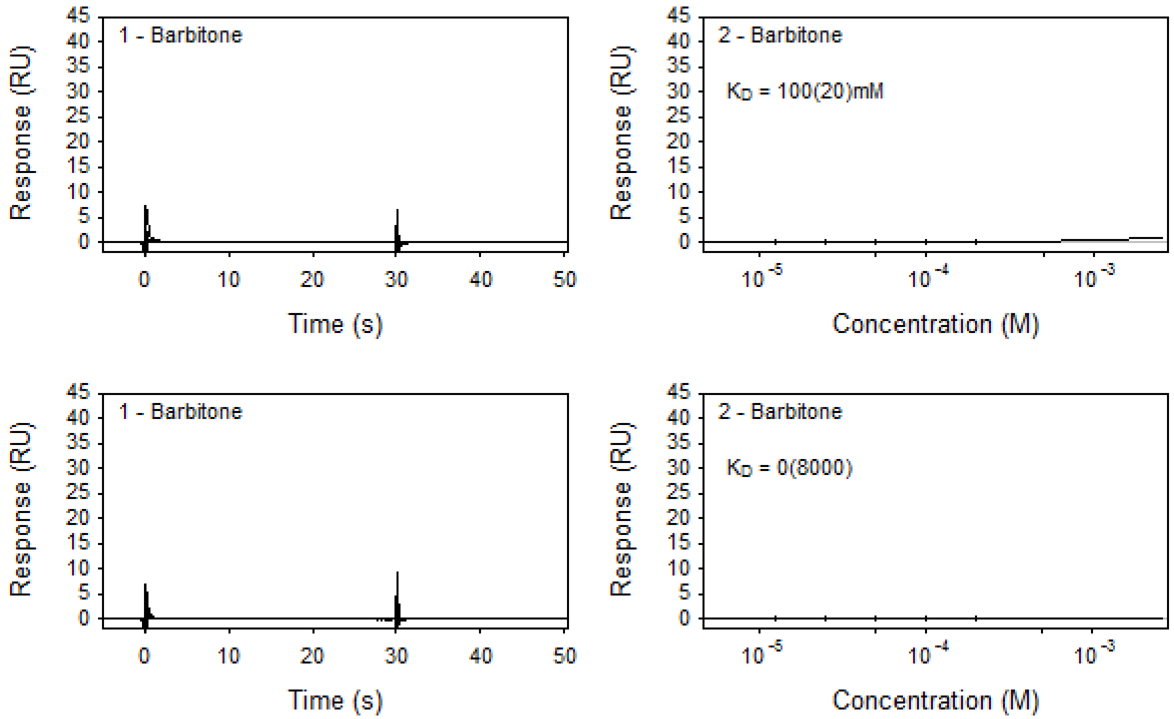

Uracil

$\chi^2 = 0.434$ RU^2

$\chi^2 = 0.0023$ RU^2

$\chi^2 = 0.0029$ RU^2

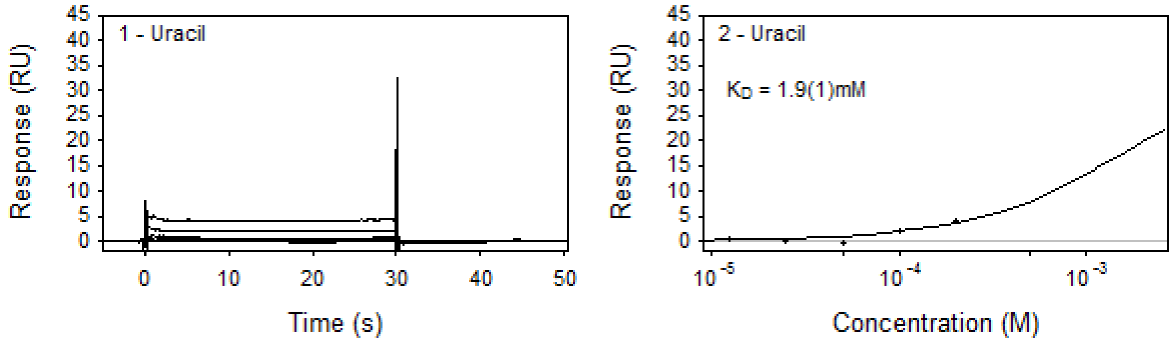

<table>
<tr>
<td></td>
<td>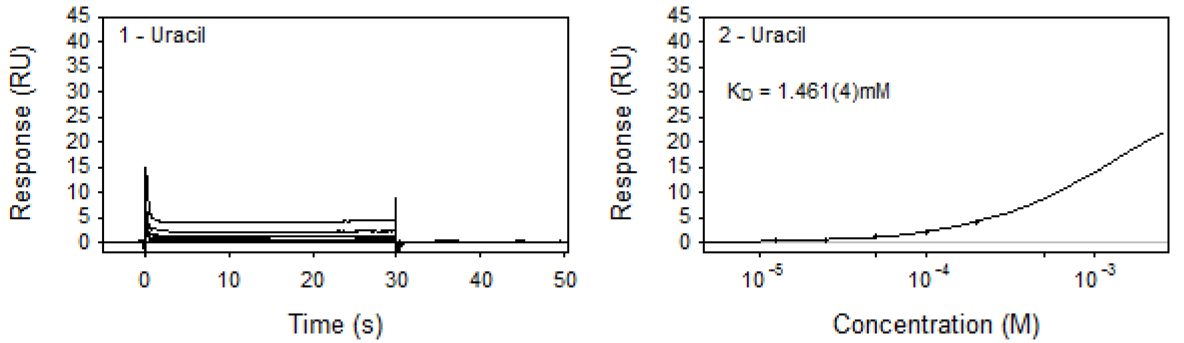

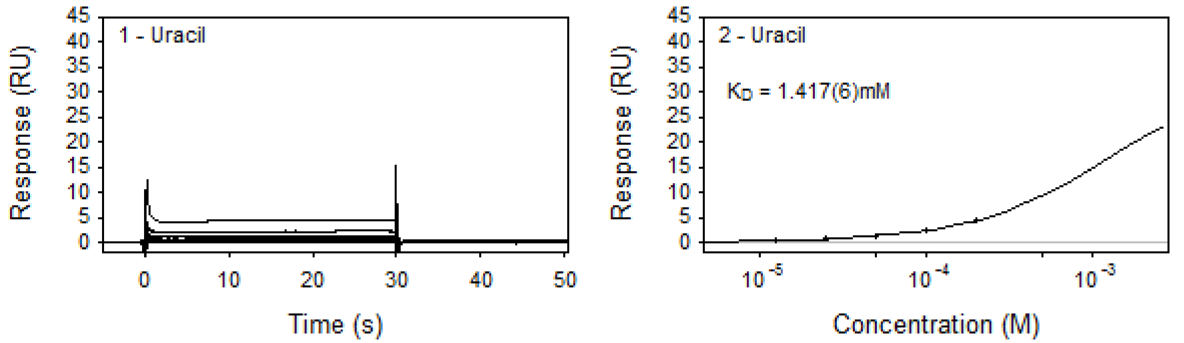
</td>
</tr>
<tr>
<td>Tetrabromoterephthalic acid

$\chi^2 = 0.0166$ RU^2
$\chi^2 = 0.0017$ RU^2
$\chi^2 = 0.0021$ RU^2</td>
<td>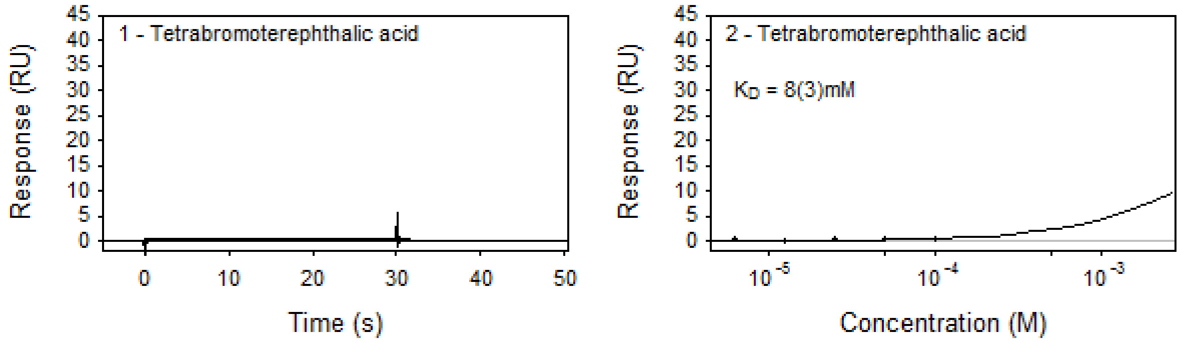
</td>
</tr>
</table>

	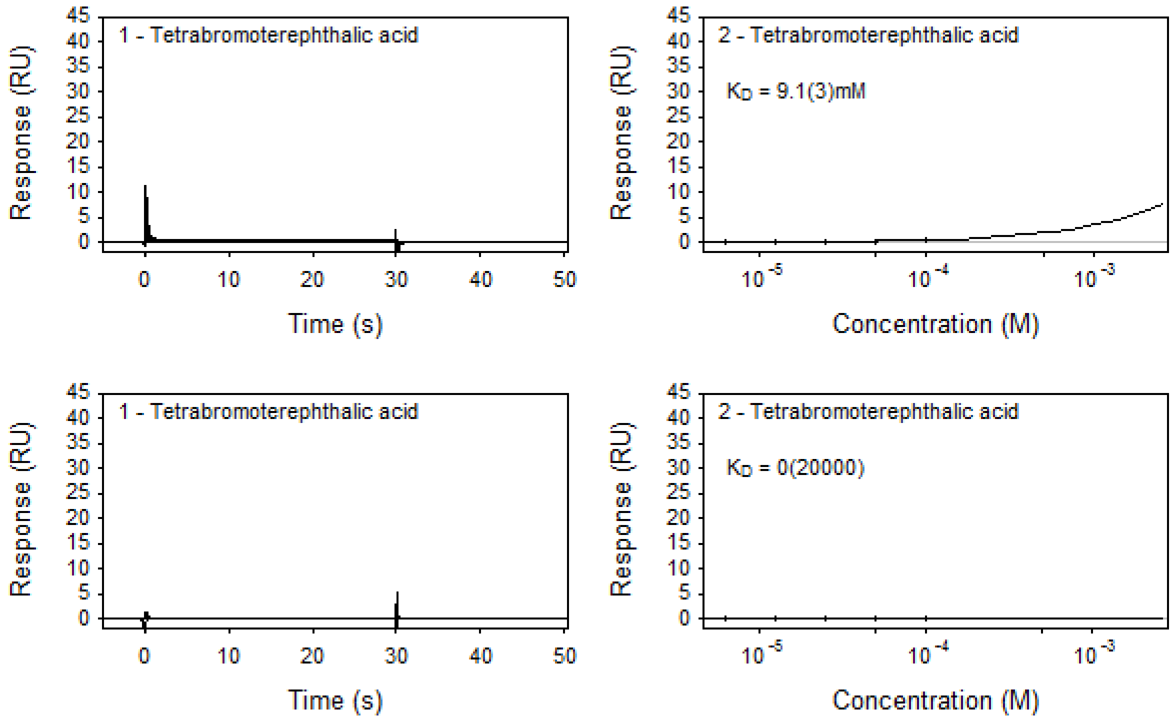
Ammeline<br><br>χ^2 = 0.0320 RU^2<br>χ^2 = 0.0278 RU^2<br>χ^2 = 0.0225 RU^2	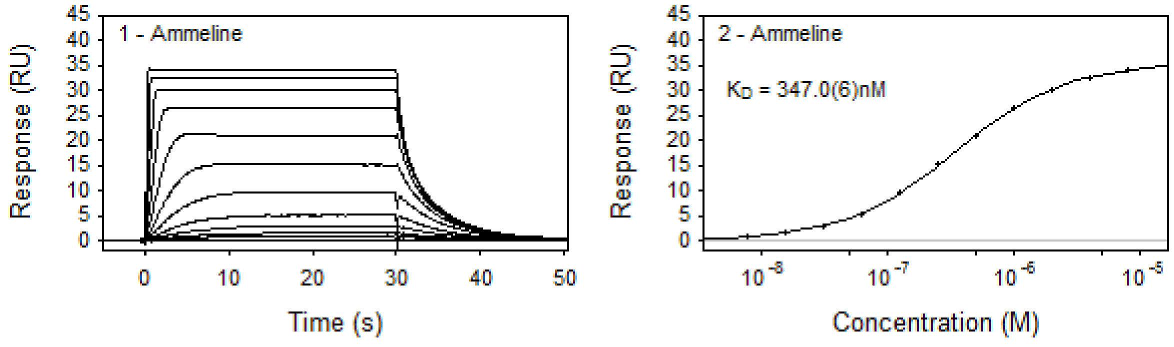

<table>
<tr><td></td><td>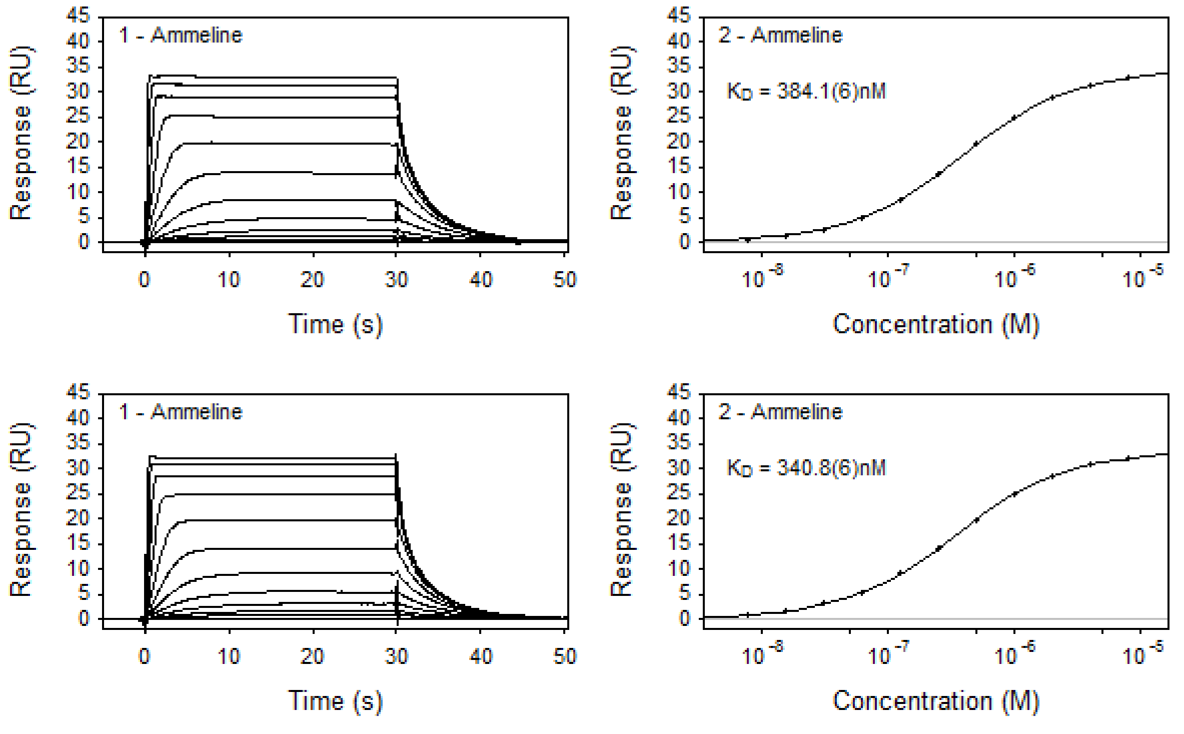
</td></tr>
<tr><td>Ammelide
Curve shape suggests a ~0.1% ammeline contaminant, based on determined ammeline affinity. Only an affinity range for ammelide (K_D > 100 µM) should be interpreted.</td><td>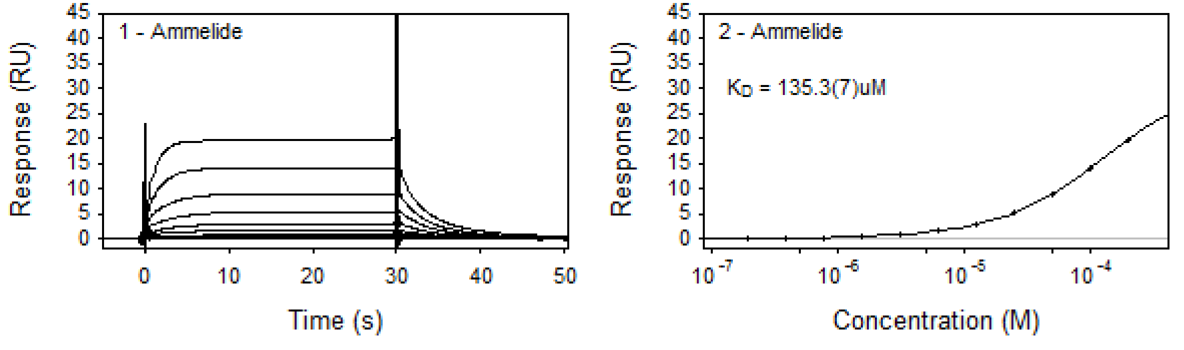
</td></tr>
</table>

<table>
<tr>
<td>$\chi^2 = 0.0046$ RU^2
$\chi^2 = 0.0659$ RU^2
$\chi^2 = 0.0536$ RU^2</td>
<td>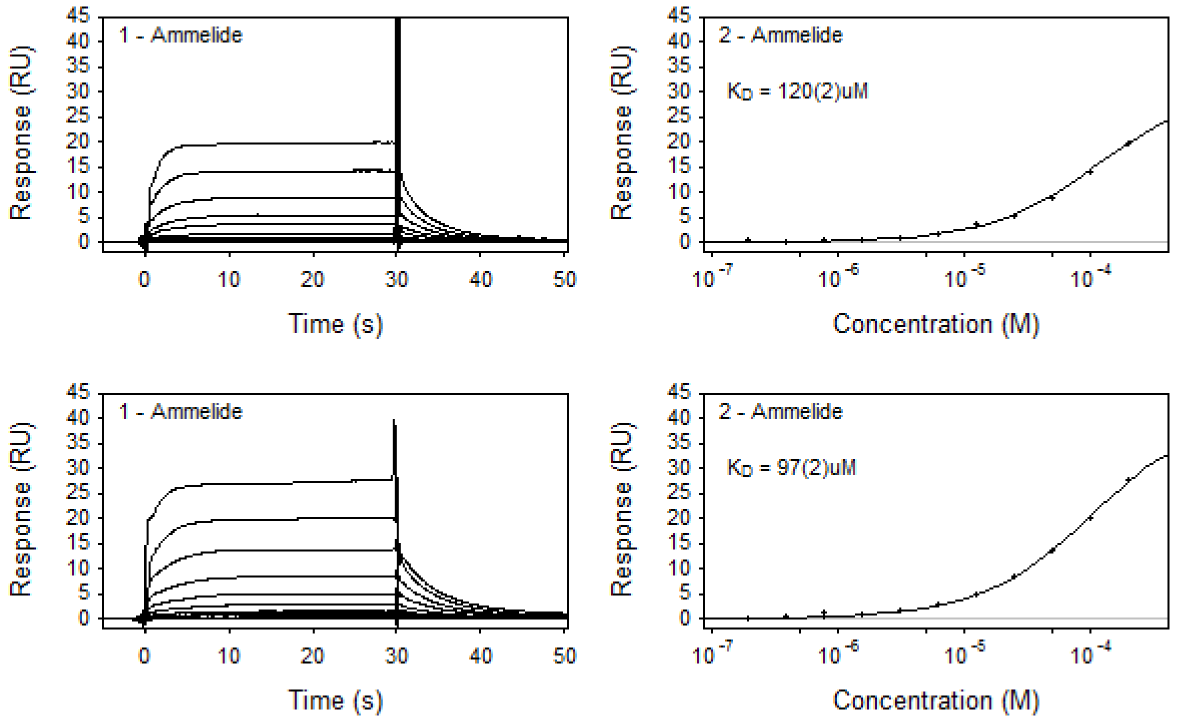
</td>
</tr>
<tr>
<td>Melamine
Curve shape suggests a <0.1% ammeline contaminant, based on determined ammeline affinity. Only an affinity range for melamine (K_D > 400 μM) should be interpreted.</td>
<td>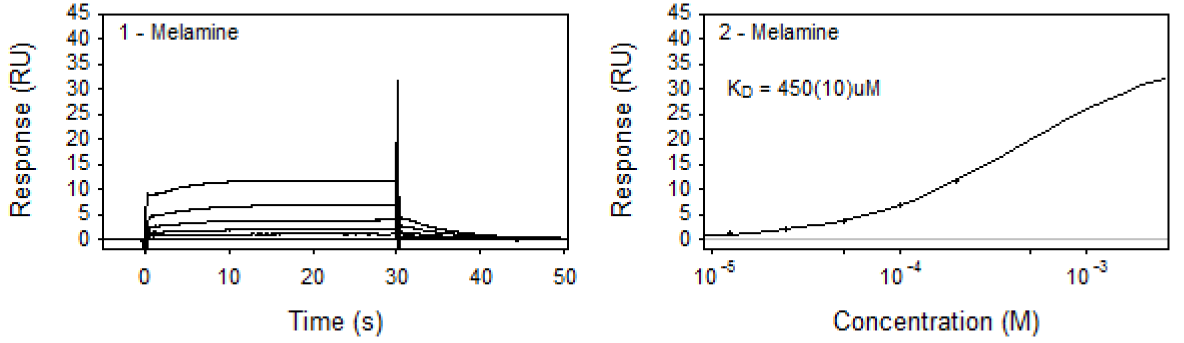
</td>
</tr>
</table>

χ^2 = 0.0034 RU^2<br>χ^2 = 0.0359 RU^2<br>χ^2 = 0.216 RU^2	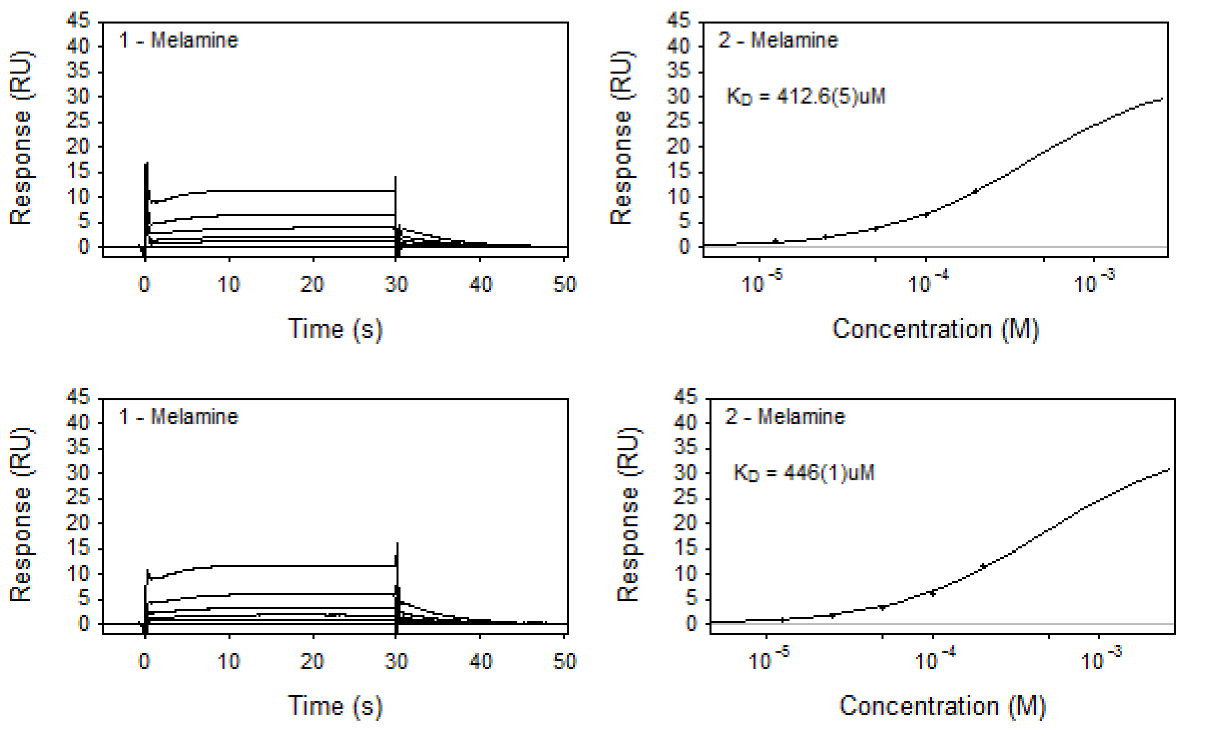
GTP<br><br>χ^2 = 0.0030 RU^2<br>χ^2 = 0.0105 RU^2<br>χ^2 = 0.0438 RU^2	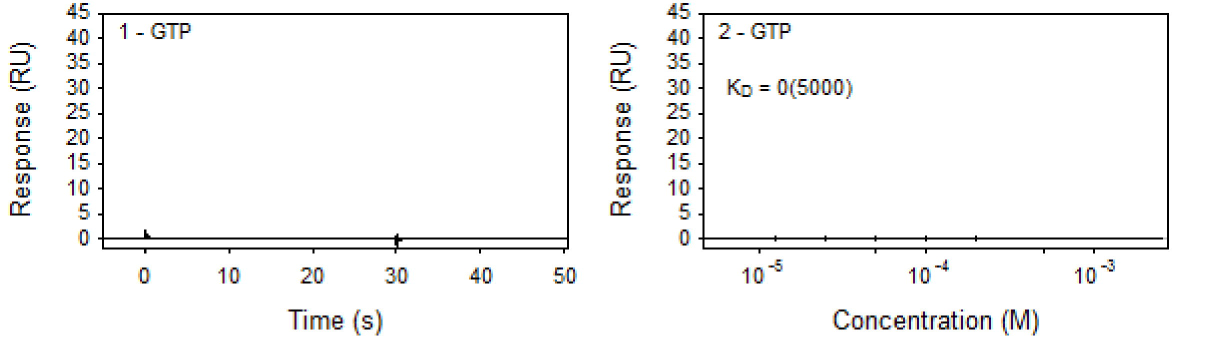

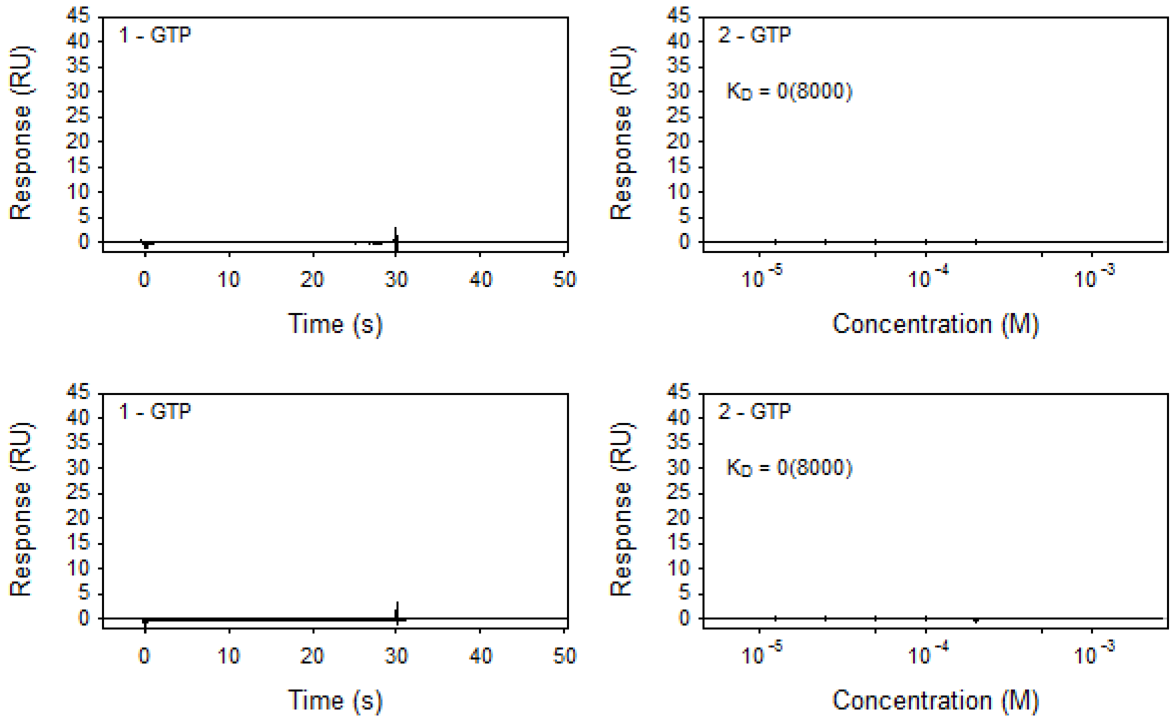
1 - GTP
Response (RU)
Time (s)
2 - GTP
K$_D$ = 0(8000)
Response (RU)
Concentration (M)
1 - GTP
Response (RU)
Time (s)
2 - GTP
K$_D$ = 0(8000)
Response (RU)
Concentration (M)

Table S1 Type and number of substrate binding proteins found in the *Pseudomonas* sp. strain ADP genome.

Type of substrate binding protein found in *Pseudomonas sp. ADP*	Number
amino acid	46
spermidine/putrescine	19
sulfur	6
polyamine/nitrate	4
metal (iron, molybdate)	7
sugar/carbohydrate	3
phosphore	3
multidrug resistance	1
unassigned SBP	23
